# Imaging articular cartilage in osteoarthritis using targeted peptide radiocontrast agents

**DOI:** 10.1371/journal.pone.0268223

**Published:** 2022-05-10

**Authors:** Milan M. Fowkes, Patricia Das Neves Borges, Fernando Cacho-Nerin, Paul E. Brennan, Tonia L. Vincent, Ngee H. Lim

**Affiliations:** 1 Centre for OA Pathogenesis Versus Arthritis, Kennedy Institute of Rheumatology, University of Oxford, Oxford, United Kingdom; 2 Diamond Light Source Ltd, Diamond House, Harwell Science and Innovation Campus, Didcot, United Kingdom; 3 Target Discovery Institute, Nuffield Department of Medicine Research Building, University of Oxford, Oxford, United Kingdom; University of Patras, GREECE

## Abstract

**Background:**

Established MRI and emerging X-ray contrast agents for non-invasive imaging of articular cartilage rely on non-selective electrostatic interactions with negatively charged proteoglycans. These contrast agents have limited prognostic utility in diseases such as osteoarthritis (OA) due to the characteristic high turnover of proteoglycans. To overcome this limitation, we developed a radiocontrast agent that targets the type II collagen macromolecule in cartilage and used it to monitor disease progression in a murine model of OA.

**Methods:**

To confer radiopacity to cartilage contrast agents, the naturally occurring tyrosine derivative 3,5-diiodo-L-tyrosine (DIT) was introduced into a selective peptide for type II collagen. Synthetic DIT peptide derivatives were synthesised by Fmoc-based solid-phase peptide synthesis and binding to *ex vivo* mouse tibial cartilage evaluated by high-resolution micro-CT. Di-Iodotyrosinated Peptide Imaging of Cartilage (DIPIC) was performed *ex vivo* and *in vivo* 4, 8 and 12 weeks in mice after induction of OA by destabilisation of the medial meniscus (DMM). Finally, human osteochondral plugs were imaged *ex vivo* using DIPIC.

**Results:**

Fifteen DIT peptides were synthesised and tested, yielding seven leads with varying cartilage binding strengths. DIPIC visualised *ex vivo* murine articular cartilage comparably to the *ex vivo* contrast agent phosphotungstic acid. Intra-articular injection of contrast agent followed by *in vivo* DIPIC enabled delineation of damaged murine articular cartilage. Finally, the translational potential of the contrast agent was confirmed by visualisation of *ex vivo* human cartilage explants.

**Conclusion:**

DIPIC has reduction and refinement implications in OA animal research and potential clinical translation to imaging human disease.

## Introduction

Plain X-ray radiography remains the gold standard for assessing cartilage damage in osteoarthritis (OA) [1]. However, it does not provide adequately sensitive disease assessment, as cartilage is a soft tissue and thus has poor X-ray absorption [1, 2]. Instead, progressive degradation of cartilage is inferred from the narrowing of space between diarthrodial joints [1]. As a consequence of this indirect measure of cartilage loss, radiography is most effective for the diagnosis of late-stage OA, once a significant proportion of cartilage has been degraded. Despite this, a considerable amount of research has focused on MRI techniques that assess the structural and biochemical composition of articular cartilage [3]. These methods can visualise early biochemical changes in cartilage degradation characterised by an increase in water content and decrease in negatively changed glycosaminoglycan (GAG) chains of proteoglycans by focusing on changes in water content (*e*.*g*. T2 mapping) or sodium ions (*e*.*g*. sodium imaging) [3]. However, the sensitive quantitation of cartilage by MRI can be limited by spatial resolution [2, 3]. Even though this can be mitigated by MRI scanners with a higher magnetic strength, these are expensive, have limited clinical availability and are poorly tolerated by elderly patients [4].

On the other hand, X-ray computed tomography (CT) possesses a superior resolution to MRI and cartilage visualisation can be enhanced with contrast agents containing radiopaque elements [2]. A number of radiocontrast agents have been used to image articular cartilage, including small molecule iodinated [5, 6] and heavy metal-containing [7–11] contrast agents. The toxicity of heavy metals limits their use to *ex vivo* imaging. Iodinated small molecules are non-specific and rely on their charge to bind to or be excluded from the negatively charged cartilage matrix. For example, electrostatic repulsion by GAGs leads to the exclusion of negatively charged compounds such as ioxaglate or iothalamate from cartilage, resulting in poor delineation at the junction between cartilage and synovial fluid [12, 13]. The advent of positively charged iodinated small molecules such as CA4+ [5] has improved uptake in cartilage and contrast delineation relative to anionic contrast agents [12–14]. However, these approaches are reliant on the fixed charge of aggrecan, which varies due to the dynamic physiological and pathological turnover of aggrecan GAGs in the OA joint [15, 16].

An alternative target for contrast agents is type II collagen in cartilage. Whilst aggrecan loss in cartilage is thought to be dynamic and reversible—as in disuse atrophy [17]—type II collagen damage is thought to be non-reversible and has direct structural and functional consequences for joint health [18]. Imaging type II collagen directly thus provides a potentially more accurate and stable picture of the disease state. We have previously shown that the type II collagen binding peptide WYRGRL [19], when conjugated to a macrocyclic DOTAM scaffold with a Cy5.5 fluorophore, successfully visualises articular cartilage *ex vivo* and *in vivo* by fluorescence imaging [20]. A more clinically relevant method to render this collagen-binding peptide radiopaque would enable the visualisation of long-term changes in articular cartilage by CT.

Herein we describe the development and validation of a radiopaque contrast agent for articular cartilage by combining a type II collagen binding peptide with the naturally occurring amino acid 3,5-diiodo-L-tyrosine (DIT) [21] using Fmoc-based solid-phase peptide synthesis (Fmoc-SPPS). We describe the generation of multiple DIT peptides based on WYRGRL, their application to *ex vivo* imaging of murine and human articular cartilage, as well as *in vivo* imaging of articular cartilage in osteoarthritic mice. To the best of our knowledge, this methodology, which we describe as Di-Iodotyrosinated Peptide Imaging of Cartilage (DIPIC), represents the first use of a collagen-targeted radiocontrast agent for monitoring osteoarthritis progression.

## Methods

### Synthesis of DIT peptide radiocontrast agents

Peptides were obtained through automated Fmoc-SPPS, purified by reverse-phase HPLC and characterised using HRMS and ^1^H-NMR. Further details and characterisation data can be found in the supporting information.

### Animal study

All animal work was conducted with institutional ethical approval according to the Animals (Scientific Procedures) Act 1986 under the Home Office project licence PPL 30/3129. C57Bl/6 mice (male, 10 weeks) purchased from Charles River (Margate, UK) were housed in individually ventilated cages, maintained under a 12-hour light/dark cycle and were allowed food and water *ad libitum* according to UK Home Office regulations. Experimental details on DMM surgery can be found in the supplemental material.

### *Ex vivo* imaging

Mice were sacrificed by inhalation of CO_2_, hind limbs collected, and tibiae finely dissected under a dissection microscope. Three tibiae per contrast agent were incubated to equilibrium in 20 mg iodine/ml and imaged by micro-CT (Quantum FX, Perkin Elmer, Waltham, Massachusetts, USA) at a resolution of 10 μm/pixel. To determine the half-life of each DIT peptide, tibiae were washed in saline and imaged upon 1 h, 3 h, 4 h, 6 h, 8 h, 24 h and 48 h of clearing in fresh saline.

### *In vivo* imaging

Mice were anaesthetised by inhalation of isoflurane and intra-articularly injected either with contrast agent in the right knee joint (20 μl, 20 mg iodine/ml) or saline (20 μl) in the left knee joint. Joints were immediately imaged by micro-CT at a spatial resolution of 10 μm/pixel (70 kV, 160 μA, 0.5 mm aluminium filter, 360° scan, 3 min acquisition time). Following imaging, animals were sacrificed by cervical dislocation. All scans were reconstructed to generate cross-sectional images using the manufacturer’s built-in software (version 2.3, PerkinElmer, Waltham, Massachusetts, USA) and subsequently resliced for analysis using ImageJ (National Institutes of Health, Bethesda, Maryland, USA), resulting in stacks of 512 isotropic voxel size images.

### Human cartilage samples

Human tissue samples and data were collected with approval from the NHS Research Ethics Committee (REC 07/H0706/81). Written informed donor consent was obtained in full compliance with national and institutional ethical requirements, the UK Human Tissue Act, and the Declaration of Helsinki (HTA License 12217). Tibial plateaus of patients undergoing knee arthroplasty were collected and osteochondral plugs (5 mm) obtained using a biopsy punch. Plugs (*n* = 2) were incubated with contrast agent for 48 h and washed with saline for 120 h. Micro-CT imaging was performed at a spatial resolution of 10 μm/pixel at multiple time points to follow tissue uptake and washout of DIT peptides.

### Patient and public involvement

Patients and members of the public were involved in several workshops run by the Centre for OA Pathogenesis to explore perceived clinical need, concept acceptability and study design.

## Results

### Design, synthesis and characterisation of DIT peptide radiocontrast agents

**Fig 1:**
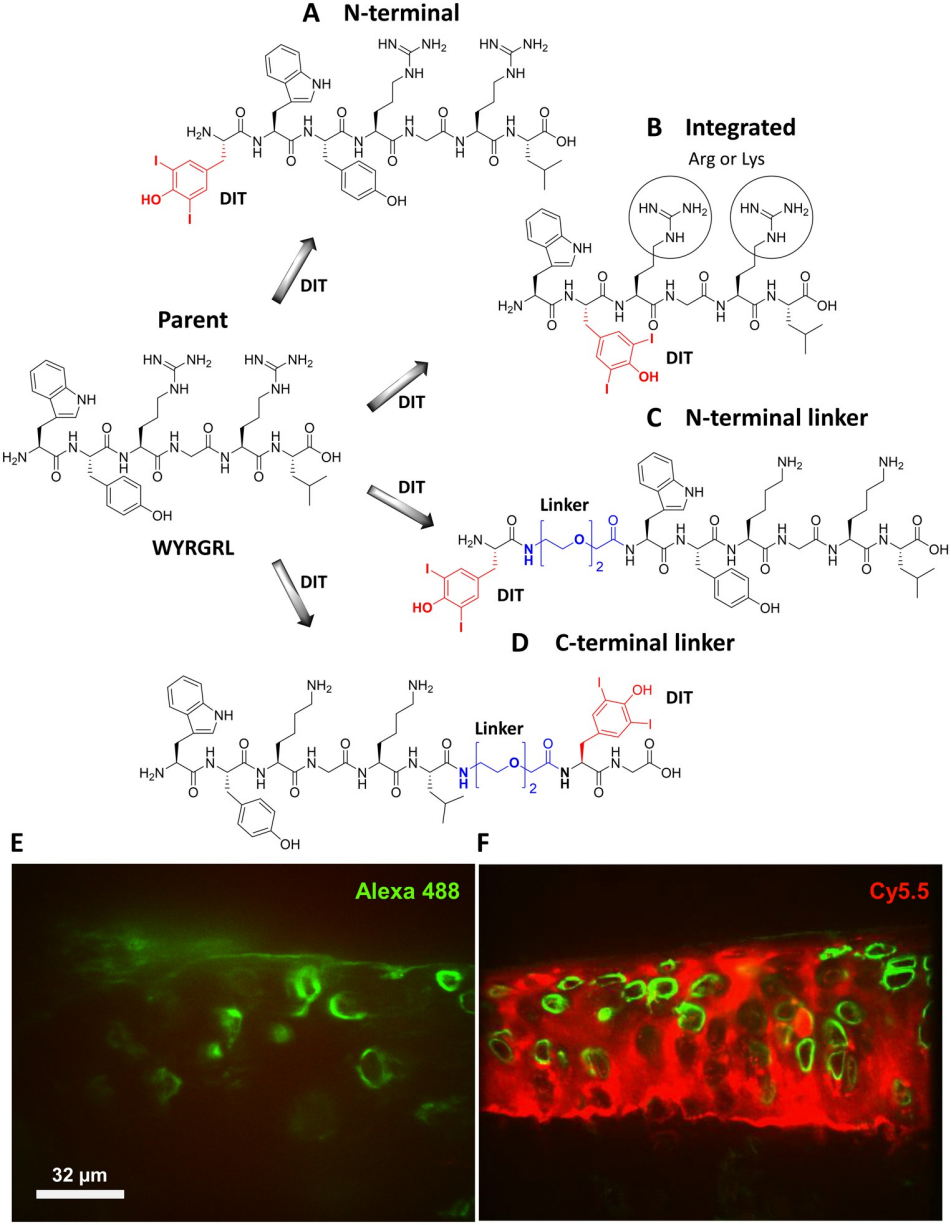
Design of targeted peptide radiocontrast agents through the insertion of DIT into the WYRGRL hexamer. Strategies include N-terminal placement of DIT (*A*), an integrated design (*B*) replacing Tyr with DIT (red) and containing Arg or Lys amino acids (circled), and an N/C-terminal PEG-based linker (*AEEA*, blue) approach to separate signalling (DIT) and targeting (WYRGRL) entities (*C*, *D*). Histology of *ex vivo* murine articular cartilage 24 h after intra-articular injection of Ac-YRLGRW-DOTAM-Cy5.5 (*E*) or Ac-WYRGRL-DOTAM-Cy5.5 (*F*) into mouse knee joints. Only targeted probe (*F*) showed binding to cartilage (Cy5.5, red) with the pericellular proteoglycan perlecan (Alexa Fluor 488, green) revealing the location of chondrocytes.

To minimise disruption to type II collagen binding, several strategies were used to introduce the DIT residue into WYRGRL (Fig 1*A*–1*D*). These involved the addition of DIT to the N-terminus of the peptide (Fig 1*A*) or utilised an integrated design, where direct replacement of Tyr was used to generate peptide sequences closely resembling WYRGRL without significantly changing its length or overall structure (Fig 1*B*). This latter design also replaced Arg for Lys to enhance water solubility. Furthermore, in order to reduce the potential impact of DIT on binding affinity when directly proximal to WYRGRL, the 8-amino-3,6-dioxaoctanoic acid (*AEEA*) linker was introduced at either the N- or C-terminus (Fig 1*C* and 1*D*, respectively). The addition of a glycine for the C-terminal design (Fig 1*D*) facilitated syntheses. Variations to these initial strategies involved combinations of integrated and C-terminal linker designs, as well as the introduction of one Lys residue for every DIT to offset the reduced solubility that DIT confers. The peptide WYRGRL was also validated as a targeting entity through injection of either scrambled control Ac-YRLGRW-DOTAM-Cy5.5 (Fig 1*E*) or targeting peptide Ac-WYRGRL-DOTAM-Cy5.5 (Fig 1*F*) into mice *in vivo* [20]. Subsequent histology revealed strong binding of the targeted probe to cartilage (Fig 1*F*) compared to the scrambled control (Fig 1*E*) [20]. All DIT peptide derivatives of WYRGRL were synthesised through automated Fmoc-SPPS, purified by reverse-phase HPLC (see S1 Fig in S1 File for chromatograms) and characterised by ^1^H-NMR spectroscopy (see S2 Fig in S1 File for spectra) and HRMS. Table 1 shows the synthesised DIT peptides and their purities.

**Table 1:** Percentage purities of synthesised DIT peptides and their *ex vivo* binding half-lives to the cartilage of murine tibiae.

Entry	Peptide Sequence	% Purity^[a]^	Half-life, h^[b]^
1	'Y'WYRGRL	NA	ND
2	'Y"Y'WYRGRL	NA	ND
3	W'Y'RGRL	≥ 98	NS
4	'Y'W'Y'RGRL	≥ 96	NS
5	W'Y'KGKL	≥ 99	2.60
6	'Y'W'Y'KGKL	≥ 97	NS
7	'Y'WGKKL	≥ 98	ND
8	K'Y'W'Y'KGKL^[c]^	≥ 99	33.86
9	K'Y'KW'Y'KGKL^[c]^	≥ 96	95.70
10	K'Y'K'Y'W'Y'KGKL^[c]^	≥ 97	298.00
11	'Y'-*AEEA*-WYKGKL	≥ 98	13.52
12	'Y"Y'-*AEEA*-WYKGKL	≥ 99	NS
13	WYKGKL-*AEEA*-'Y'G	≥ 99	4.95
14	W'Y'KGKL-*AEEA-*'Y'G	≥ 99	NS
15	K'Y'K'Y'-*AEEA*-WYKGKL	≥ 99	93.80

'Y' = 3,5-diiodo-L-tyrosine.

^[a]^ Purity was established by integration of the area under the curve for each HPLC chromatogram at all wavelengths.

^[b]^ Murine tibiae were incubated with contrast agent, washed with saline and then imaged in saline until signal was no longer detected.

^[c]^ These peptide sequences began to form viscous gels on dissolution, so this strategy was not pursued further. NA: only crude peptide tested. ND: no visible binding to cartilage by micro-CT. NS: crude peptide bound but was insoluble in 10% DMSO/water at ≥ 95% purity by HPLC at the minimum concentration of 2 mg iodine/ml required for radiocontrast detection.

### *Ex vivo* binding of DIT peptide radiocontrast agents to murine articular cartilage

Initially, the concentration of iodine required for effective contrast delineation between cartilage, background saline and cortical bone was found to be 20 mg iodine/ml using the contrast agent diatrizoic acid (S3 Fig in S1 File). To screen multiple sequences rapidly, peptides were initially synthesised and tested without further purification. If binding to cartilage was observed, they were synthesised, purified by HPLC (≥ 95%) and re-tested to confirm the initial result. Freshly dissected murine tibiae were equilibrated with each DIT peptide (20 mg iodine/ml) and imaged by micro-CT. To determine the binding half-life, tibiae were washed with saline and imaged at regular time points until radiocontrast signal was no longer detected. The *ex vivo* half-life (t_1/2_) of each DIT peptide is displayed in Table 1, with the corresponding decay curves in S4*A* Fig in S1 File.

The addition of one or two *N*-terminal DIT residues to WYRGRL (Table 1, entries **1, 2**) revealed no observable binding to cartilage during micro-CT imaging. An integrated design, placing DIT into the core of WYRGRL (**3**) was followed by the addition of a further DIT at the N-terminus (**4**). Despite both peptides binding at the crude level, the result could not be replicated with pure peptides due to poor solubility at 20 mg iodine/ml as well as 2 mg iodine/ml; the lowest concentration required for contrast generation (S3 Fig in S1 File). To improve solubility, both arginine residues in **3** and **4** were replaced with lysine to give **5** and **6**, respectively. This delivered the first peptide with a measurable half-life (entry **5**, Table 1, t_1/2_ = 2.60 h). The scrambled peptide **7** did not show binding, revealing the importance of sequence order for successful interaction with type II collagen in cartilage. Unfortunately, peptide **6** was insoluble in solution. For this reason, further N*-*terminal addition of DITs alone was abandoned. Instead, a lysine residue was introduced for every DIT to improve solubility, increase the number of positively charged side-chains and improve overall detection sensitivity by increasing the percentage iodine content within each sequence (entries **8**–**10**, t_1/2_ = 33.86 h, 95.70 h and 298.00 h, respectively). These peptide sequences began to form viscous gels on dissolution, so this strategy was not pursued further.

In lieu of multiple lysine residues, the *AEEA* linker strategy was employed. The separation of WYRGRL from directly bound DITs was considered favourable for cartilage binding (entries **11–14**). This was found to be the case for both N- and C-terminal addition; peptide **11** (t_1/2_ = 13.52 h) and **13** (t_1/2_ = 4.95 h), respectively. The addition of more than one DIT resulted in insoluble peptide sequences at purities ≥ 95% by HPLC (**12, 14**). However, this was negated by the addition of N-terminal lysine residues into **12** to give **15** (t_1/2_ = 93.80 h).

These results show that not only can targeted peptide radiocontrast agents be generated through insertion of DIT into WYRGRL, but their *ex vivo* half-life may also be tuned to suit a particular time window in CT imaging experiments. For example, the half-life obtained for **5** was 2.60 h, which was modulated to 298.00 h for **10** by the addition of two DIT and lysine residues apiece.

To explore alternative methods to tune the *ex vivo* half-life of DIT peptides, different concentrations of iodine were utilised for peptide **11** (S4*B* Fig in S1 File). At low concentrations (5 mg iodine/ml), a rapid washout time from cartilage was observed (t_1/2_ = 1.45 h). Increasing the concentration to 10 mg iodine/ml resulted in an approximately 8-fold increase in washout time (t_1/2_ = 11.40 h). However, at 20 mg iodine/ml, only an incremental 1.2-fold increase in washout time was observed (t_1/2_ = 13.52 h). At 35 mg iodine/ml, a similar washout time was observed to 20 mg iodine/ml (*cf*. t_1/2_ = 13.46 h to t_1/2_ = 13.52 h for 20 mg iodine/ml). This similarity in half-life presumably represents the saturation limit of articular cartilage; the *ex vivo* binding half-life can no longer increase as all cartilage binding sites of **11** are occupied. Therefore, 20 mg iodine/ml is also likely to be a good working concentration to maximise signal detection through saturation of cartilage binding.

**Fig 2:**
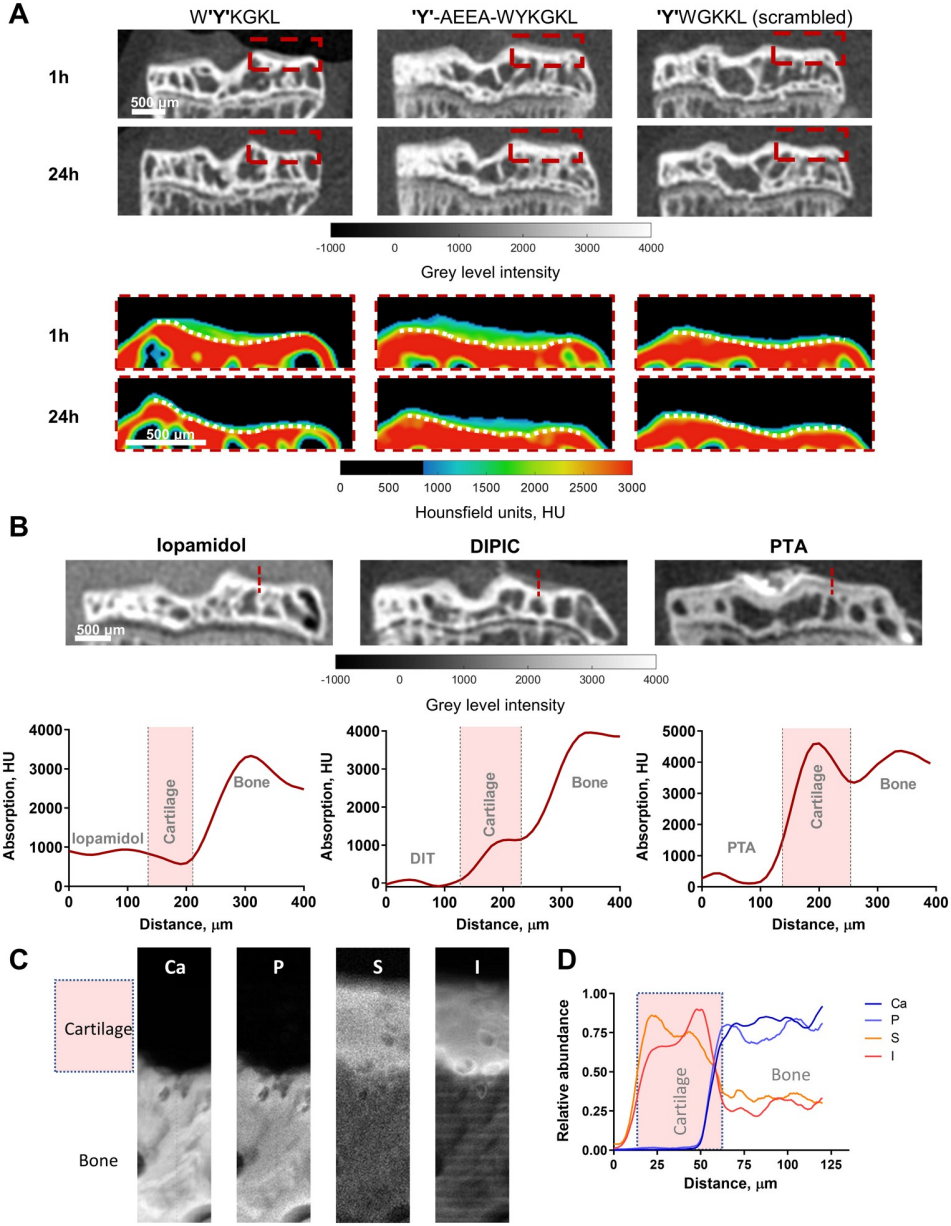
*Ex vivo* imaging of healthy murine articular cartilage using DIPIC and other radiocontrast agents. (*A*) Representative micro-CT coronal views of mouse tibiae incubated with W**'Y'**KGKL, **'Y'**-*AEEA*-WYKGKL and **'Y'**WGKKL upon 1 h and 24 h of washing in saline. The colour-coded maps of absorption (HU) for the lateral tibial plateau (red dotted box) highlight the loss of contrast enhancement as probes are cleared from cartilage over time. The dotted white lines delineate cartilage from bone. (*B*) Representative micro-CT coronal views of mouse tibiae after overnight incubation with iopamidol, **'Y'**-*AEEA*-WYKGKL (both at 20 mg iodine/ml), and PTA (1% PTA in 70% ethanol). X-ray absorption profiles for each contrast agent were generated by drawing a line across cartilage from probe to bone. (*C*) X-ray fluorescence images for *ex vivo* murine articular cartilage after incubation with **'Y'**-*AEEA*-WYKGKL showing calcium, phosphorous, sulphur and iodine channels (500 nm/pixel). (*D*) Elemental fluorescence profiles were generated by drawing a line down the middle of each image from (*C*).

The most successful DIT peptide insertion strategies involved integrated and 8-amino-3,6-dioxaoctanoic acid (*AEEA*) linker designs. Fig 2*A* shows representative *ex vivo* micro-CT coronal images of mouse tibiae incubated with peptides from these strategies (*i*.*e*. W**'Y'**KGKL, **5**, **'Y'**-*AEEA*-WYKGKL, **11** as well as the scrambled control for one of these sequences; **'Y'**WGKKL, **7**) upon 1 h and 24 h of washing in saline. Articular cartilage can be observed on both the medial and lateral tibial plateau of **5** and **11** after 1 h of washing (a time point in which unbound peptide has cleared from surrounding tissues apart from cartilage), but no cartilage is visible for **7**. After 24 h, complete washout was observed for peptide **5**, whereas for peptide **11** some binding could still be visualised (t_1/2_ = 13.52 h). The colour-coded maps of X-ray absorption (expressed in Hounsfield units, HU) of the lateral aspect of the tibial plateau for each peptide (expanded red box) highlight the difference in contrast between bone, cartilage, and saline, as well as the clearance of each peptide from cartilage.

### *Ex vivo* micro-CT imaging of murine articular cartilage using DIPIC, phosphotungstic acid (PTA) and iopamidol

To validate the degree of contrast enhancement of murine articular cartilage by DIPIC, **'Y'**-*AEEA*-WYKGKL was compared with pre-clinical (PTA) and clinical (iopamidol) radiocontrast agents (Fig 2*B*). A profile of tissue X-ray absorption showed that iopamidol exhibited poor contrast enhancement of cartilage, with a 0.8-fold difference (mean ± SEM) in absorption between cartilage and background (*cf*. 678 ± 29 HU to 875 ± 12 HU). In contrast, DIPIC revealed a 7.2-fold difference in absorption between cartilage and background (*cf*. 690 ± 103 HU to 96 ± 17 HU), clearly delineating cartilage from the surrounding solution. A higher (10.7-fold) contrast difference between cartilage and background was observed using PTA (*cf*. 3657 ± 253 HU to 342 ± 64 HU). Both DIPIC and PTA assessed the thickness of cartilage similarly (*cf*. 100 μm to 110 μm), while iopamidol gave a lower value of 70 μm, indicating a lower detection sensitivity compared to DIPIC at 20 mg iodine/ml. DIPIC thus provides similar delineation between cartilage and background to PTA and is approximately ten times better than iopamidol, a clinically approved radiocontrast agent.

We tested the ability of DIPIC to detect lesions in murine articular cartilage by puncturing the articular surface of tibiae using needles with different outer diameters (O.D.) (S5 Fig in S1 File). The resulting damage (indicated by red arrows) ranged from small clefts (O.D. of 184.2 μm), to focal cartilage loss (O.D. of 412.8 μm), to extensive abrasion of the surface (O.D. of 514.4 μm). Remarkably, DIPIC was able to visualise the presence of these increasingly larger lesions whilst also imaging articular cartilage in an undamaged sample. To verify the depth of penetration of DIT peptides into articular cartilage, **'Y'**-*AEEA*-WYKGKL was incubated with murine articular cartilage and subjected to X-ray fluorescence (XRF) analysis. Elemental analysis of calcium and phosphorous revealed the mineral bone, sulphur the proteoglycans in cartilage, and iodine the DIT peptide (Fig 2*C*). In the iodine channel, **'Y'**-*AEEA*-WYKGKL was found to surround the chondrocytes in the pericellular matrix near the deep zone of articular cartilage. The relative abundance of each element is shown in Fig 2*D*.

### *Ex vivo* micro-CT imaging of articular cartilage in osteoarthritic mice using DIPIC and validation using PTA-CT

**Fig 3:**
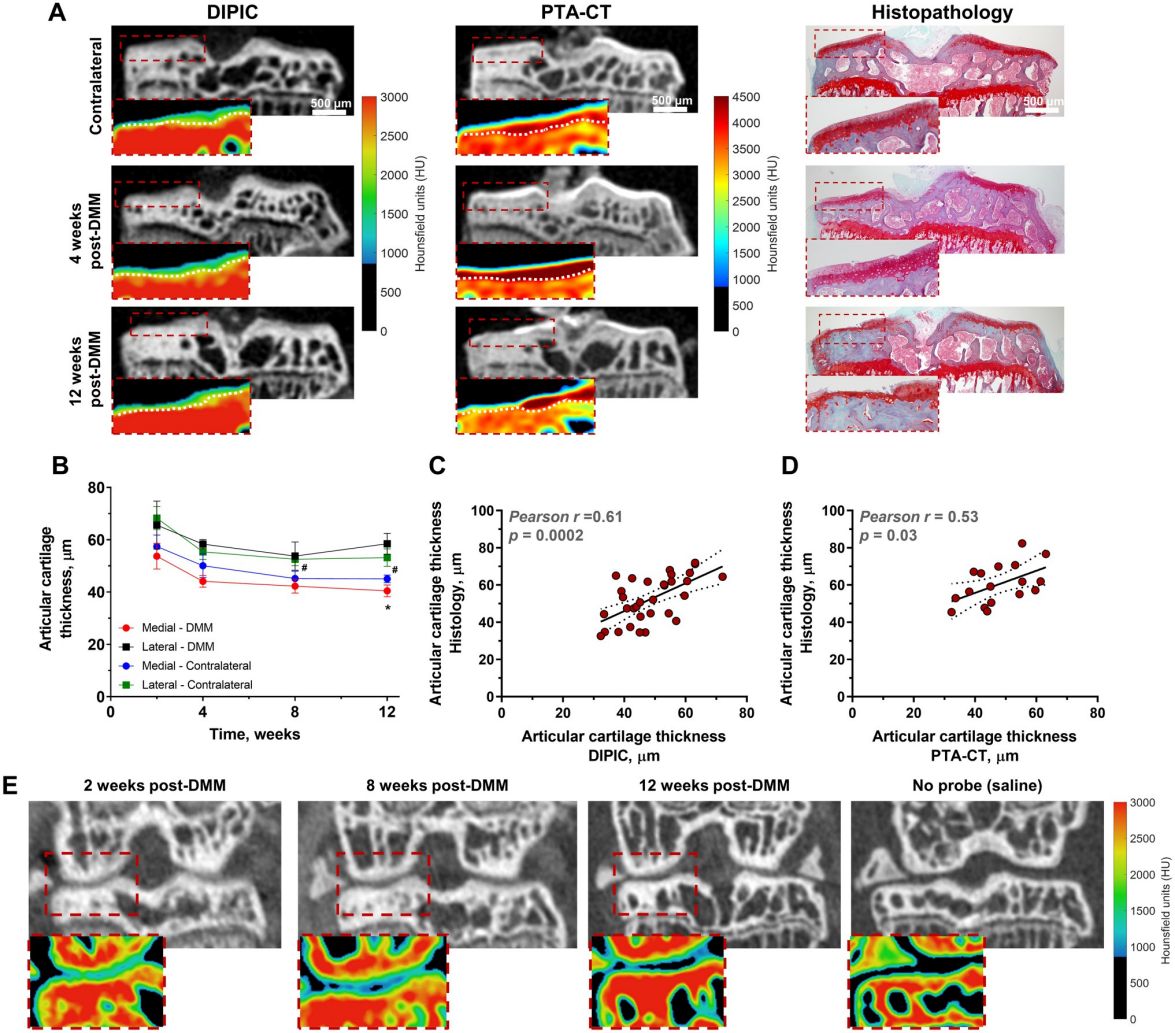
*Ex vivo* and *in vivo* imaging of murine osteoarthritis using DIPIC. (*A*) Representative micro-CT coronal views of contralateral tibia (negative control) and operated tibiae at 4 weeks and 12 weeks post-surgery imaged using DIPIC and subsequently PTA-CT. Despite PTA-CT providing higher contrast enhancement of cartilage than DIPIC (see colour-coded maps of absorption), both methods revealed the progressive degradation of this tissue in the medial tibial plateau (dotted red boxes). Dotted white lines delineate cartilage from bone. The presence of lesions in DMM mice was validated by histopathology (haematoxylin & eosin and safranin-O staining in the far-right panel). (*B*) Average articular cartilage thickness in DMM-operated and contralateral tibiae in weeks post-DMM surgery. Mean ± SEM, *p < 0.05, obtained by one-way ANOVA with Dunnett’s multiple comparison post-test for differences between 2 weeks vs. subsequent time points in the medial DMM tibial plateau and ^#^*p* < 0.05 for the same test in the medial contralateral tibial plateau. Parametric correlations between articular cartilage thickness measurements obtained from histological sections and by DIPIC (*C*) (*n* = 32) and PTA-CT (*D*) (*n* = 17); Pearson correlation coefficients (*r*) and *p*-values are indicated in the graph. (*E*) Representative micro-CT coronal views of DMM mouse knee joints and corresponding colour-coded maps of absorption of the medial aspect of the joint after injection of either **'Y'**-*AEEA*-WYKGKL or saline into the synovial space. Progressive cartilage degradation is highlighted by DIPIC, but not by saline, owing to the loss of contrast enhancement.

The ability of DIPIC to track and quantify progressive articular cartilage degradation was tested using a surgical model of murine osteoarthritis involving destabilisation of the medial meniscus (DMM) [22]. Tibia dissected from DMM mice were incubated with **'Y'**-*AEEA*-WYKGKL at different time points following disease induction and DIPIC visualised gradual cartilage degradation during disease progression (Fig 3*A*). DIPIC further presented sufficient contrast to automatically delineate cartilage from bone (S6*A* and S6*B* Fig in S1 File), enabling assessment of disease progression across the entire tibial surface. Cartilage thinning was evident in the medial tibial plateau of operated mice and was detected by DIPIC at time points in which disease was already well established (Fig 3*B*), with histopathology confirming the presence of osteoarthritic lesions in cartilage (Fig 3*A*, far right panel). Manual segmentation of articular cartilage in histological sections and subsequent quantification of thickness revealed a clear correlation between cartilage thickness assessed by histology and that determined by DIPIC and PTA-CT (Fig 3*C* and 3*D*). The former showed a high (*r* = 0.61) and significant (p = 0.0002) correlation with histology (Fig 3*C*). Measurements in 3D for DIPIC and PTA-CT were then obtained by approaches based on automated segmentation and mapping of volumes-of-interest (VOIs). A comparison between both indicated that they were significantly correlated (S6*C* Fig in S1 File) and in good agreement (S6*D* and S6*E* Fig in S1 File). Compared to PTA-CT, DIPIC quantifications appeared to underestimate cartilage thickness by 7.1 μm (S6*E* Fig in S1 File) an amount below the resolution limit of micro-CT (10 μm/pixel). Furthermore, the medial and lateral cartilage thickness of DMM-operated tibiae was assessed by DIPIC and compared to OARSI histopathological scoring (S6*F* and *S6G* Fig in S1 File). A clear negative correlation was observed between medial cartilage thickness and OARSI scoring (*p* = 0.0002), but not for lateral cartilage. Thus, higher scores on the medial side of tibiae were associated with thinner cartilage.

### *In vivo* micro-CT imaging of articular cartilage in osteoarthritic mice using DIPIC

To test prospective monitoring of cartilage loss, an intra-articular injection of **'Y'**-*AEEA*-WYKGKL was given to a group of mice every week up to 12 weeks after disease induction and was rapidly followed by *in vivo* imaging of the knee joint (Fig 3*E*). A saline injection into the contralateral knee acted as a negative control and animals were rightly sacrificed upon imaging. Articular cartilage can clearly be evinced in knees containing DIT peptide, whereas no cartilage is observed in the knee where saline was injected; the colour-coded maps of absorption of the medial aspect of the joint (red dashed boxes) confirm these observations. At early timepoints after disease induction (two weeks post-DMM), undamaged cartilage is visualised by DIPIC at the margins of bone. However, at 8-weeks post-DMM cartilage is seen to be thinning in the load-bearing area. This is further enhanced at 12 weeks post-DMM, an observation which is consistent with *ex vivo* quantitation (Fig 3*B*). The negative control confirms that saline solution does not result in cartilage visualisation. DIPIC with **'Y'**-*AEEA*-WYKGKL may thus be used to visualise articular cartilage in mice *in vivo*.

### *Ex vivo* micro-CT imaging of human articular cartilage using DIPIC

**Fig 4:**
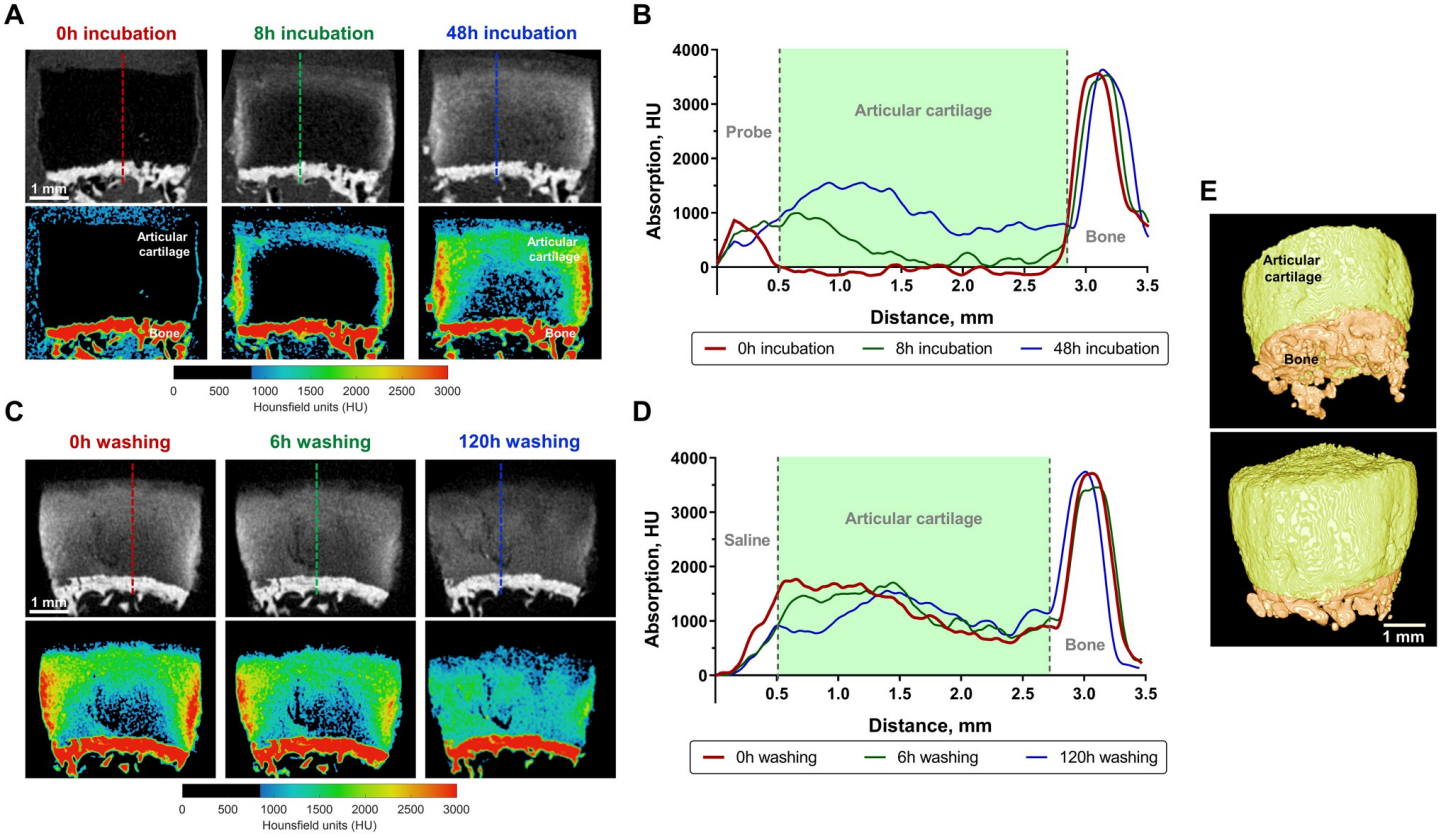
*Ex vivo* imaging of human articular cartilage with the DIT peptide 'Y'-*AEEA*-WYKGKL. (*A*) Representative micro-CT views of a human osteochondral plug at different time points upon incubation with probe and corresponding colour-coded maps of absorption. (*B*) X-ray absorption profiles (dashed coloured lines in micro-CT images) obtained to evaluate probe penetration into tissue over time. (*C*) Representative micro-CT views of the same osteochondral plug at different time points upon washing in saline and corresponding colour-coded maps of absorption. (*D*) X-ray absorption profiles (dashed coloured lines in micro-CT images) obtained to evaluate probe washing from tissue over time. (*E*) Representative views of a 3D reconstruction of the osteochondral plug showing articular cartilage imaged by the DIT peptide (green) and underlying subchondral bone.

Human osteochondral plugs were immersed in **'Y'**-*AEEA*-WYKGKL and imaged by micro-CT throughout incubation. A gradual diffusion of peptide was observed into the plug over time (Fig 4*A*, upper row). However, the deeper zones of cartilage had lower uptake of peptide (Fig 4*A*, lower row) and thus lower X-ray absorption (Fig 4*B*). The bound peptide was washed out with saline solution up to 120 h (Fig 4*C*, upper row). Colour-coded maps of absorption (Fig 4*C*, lower row) and profiles generated across the sample (Fig 4*D*) showed that peptide remained in the tissue five days after saline washing. At this time point, greater uptake was observed in the deeper zones with more uniform distribution throughout cartilage. A 3D reconstruction of the osteochondral plug revealed that articular cartilage and underlying bone could be clearly visualised using this probe (Fig 4*E*). These results demonstrate that DIPIC can be successfully used for *ex vivo* 3D imaging of articular cartilage from human biopsies.

## Discussion

In this paper, we describe the development of targeted peptide radiocontrast agents to monitor articular cartilage degradation in osteoarthritis. We validated this *ex vivo* in murine and human articular cartilage as well as *in vivo* in osteoarthritic mice. *Ex vivo*, DIPIC demonstrated a ten-fold improvement in contrast enhancement compared with the clinical contrast agent iopamidol, and enhancement correlated well with data obtained using PTA, a toxic *ex vivo* contrast agent. In addition, human cartilage was also readily visualised. *In vivo*, DIPIC was able to visualise articular cartilage damage in OA mice following intra-articular injection. The sensitivity of this as an imaging tool was demonstrated by showing a good correlation between DIPIC signal and histological cartilage grade in OA mice, and by showing that small superficial defects (induced by scarification of the cartilage) could also be picked up by this agent.

Compared with the half-life of aggrecan in articular cartilage (3.4 years) [23], type II collagen has a half-life of 117 years [24], making it a more stable imaging target for longitudinal OA studies. We refined the type II collagen binding peptide WYRGRL [19] with respect to cartilage binding by inserting DIT through several strategies, thereby generating probes with a range of washout half-lives from *ex vivo* murine tissue. We did not observe adverse events during the study, although the *in vivo* studies were short (< 10 min) and performed under terminal anaesthesia. As DIT is a naturally occurring amino acid [21], its use in DIPIC is unlikely to have direct toxic effects when compared with a heavy metal. Nonetheless, future prospective *in vivo* studies will require a detailed examination of cellular toxicity.

Our study shows that DIPIC has potential value as a pre-clinical monitoring tool. Murine OA induced by DMM replicates many features of the human condition, including slow progressive cartilage degradation, bone remodelling, modest synovitis and late onset pain behaviour [22, 25–27]. Our standard histological processing involves serial sectioning through the entire depth of the joint with double-blind scoring performed on at least eight stained sections per animal at a minimum of two time points after surgery. An *in vivo* tool to monitor structural disease progression in mice would save significant time and money and provide a clearer understanding of the stages of disease progression. It could also be used to select the most appropriate time for termination of experiments as well as monitor treatment responses and dose finding. Taken together, these benefits would have significant impact on the reduction of mouse numbers in OA research in line with the ARRIVE guidelines [28] and be more relevant for human clinical use. Our *in vivo* data will require further refinement to deliver the benefits above. This could include increasing the signal from the collagen binding peptide, improving the distribution of contrast agent following injection and/or improving image registration in live animals.

Whilst our study was largely focused on developing an *in vivo* imaging tool to support pre-clinical OA research, our findings also have potential application to human disease monitoring. There is a pressing need to develop diagnostic, prognostic and theragnostic radiographic biomarkers that are more sensitive than standard X-ray. A number of contrast agents have been tested experimentally in human studies. These are largely MRI-based due to the superior safety of MRI over CT [2]. MRI-based cartilage imaging has largely utilised contrast media targeting the fixed charge density of aggrecan such as delayed gadolinium-enhanced magnetic resonance imaging of cartilage (dGEMRIC) [29, 30] or through sodium imaging [31, 32]. Multimodal techniques incorporating MRI with Positron Emission Tomography (PET) are also being explored [33, 34]. However, despite MRI having a superior safety profile, it is expensive, suffers from long acquisition times and is frequently not tolerated by patients due to its claustrophobic nature [2].

We recognise a number of limitations in our study. Firstly, the WYRGRL binding site on type II collagen is not fully characterised, and we are unclear whether binding (and therefore signal) would be affected by post-translational changes in type II collagen that occur with age and disease *e*.*g*. through cross-linking or damage due to reactive oxygen species [35]. Nor do we know whether the accessibility of the binding site would be affected by changes in other matrix proteins such as loss of aggrecan following proteolytic degradation. Secondly, collagen itself binds to cell surface integrins and is able to activate intracellular signalling in chondrocytes through discoidin domain receptors (DDRs) [36]. Whether the WYRGRL peptide interferes with the generation of native triple helical fragments or blocks binding at the receptor, is unknown. Although it is unlikely that WYRGRL would activate DDRs directly, as it does not have a triple helical structure. Finally, our study was limited by the resolution of our micro-CT scanner (10 μm/pixel) as this scanner is primarily designed for *in vivo* use.

## Conclusions

To conclude, we have developed a novel and versatile system for labelling specific extracellular matrix proteins *in vivo*. For disease monitoring, DIPIC could be used alone or in conjunction with other imaging modalities such as PET. Its immediate use may be in pre-clinical studies to improve the time and cost associated with tissue processing, but it also possesses potential as a clinical radiographic biomarker, supporting clinical studies and ultimately assisting in the clinical management of patients.

## Supporting information

S1 File (DOCX)
