# Supplementary material for: Imaging articular cartilage in osteoarthritis using targeted peptide radiocontrast agents
Source: PLoS One. 2022 May 10;17(5):e0268223. doi: 10.1371/journal.pone.0268223 (PMC9089912; doi:10.1371/journal.pone.0268223)
Supplement: S1 File — (DOCX) [file pone.0268223.s001.docx]

Supporting information

Imaging articular cartilage in osteoarthritis using targeted peptide radiocontrast agents

Milan M. Fowkes,^1¶^ Patricia Das Neves Borges,^1¶^ Fernando Cacho-Nerin,^2^ Paul E. Brennan,^3^ Tonia L. Vincent^1^ and Ngee H. Lim^1^*

^1^ Centre for OA Pathogenesis Versus Arthritis, Kennedy Institute of Rheumatology, University of Oxford, Roosevelt Drive, Headington, Oxford, OX3 7FY, United Kingdom

^2^ Diamond Light Source Ltd, Diamond House, Harwell Science and Innovation Campus, Didcot, OX11 0DE, United Kingdom

^3^ Target Discovery Institute, Nuffield Department of Medicine Research Building, University of Oxford, Old Road Campus, Headington, Oxford, OX3 7FZ, United Kingdom

^¶^ These authors contributed equally to this work.

**Table of Contents**

[Methods 3](#_Toc102162543)

[Peptide chemistry 3](#_Toc102162544)

[Peptide synthesis 3](#_Toc102162545)

[Peptide cleavage and purification 3](#_Toc102162546)

[Characterisation of DIT peptides 4](#_Toc102162547)

[Micro-CT imaging 8](#_Toc102162548)

[General information 8](#_Toc102162549)

[Iodine calibration curve 9](#_Toc102162550)

[Animal study 9](#_Toc102162551)

[Murine articular cartilage 9](#_Toc102162552)

[Fluorescence immunohistochemistry for localisation of Ac-WYRGRL-DOTAM-Cy5.5 9](#_Toc102162553)

[Calculation of *ex vivo* DIT peptide half-life 9](#_Toc102162554)

[X-ray fluorescence imaging of *ex vivo* murine articular cartilage 10](#_Toc102162555)

[*Ex vivo* imaging with DIPIC, PTA or iopamidol 10](#_Toc102162556)

[Generation of colour-coded absorption maps 11](#_Toc102162557)

[Surgical induction of OA through destabilisation of the medial meniscus (DMM) 11](#_Toc102162558)

[*In vivo* imaging in DMM mice using DIPIC 11](#_Toc102162559)

[*Ex vivo* quantitative imaging of DMM mice using PTA and DIPIC 12](#_Toc102162560)

[Histopathology of DMM mice 12](#_Toc102162561)

[Statistical analysis 13](#_Toc102162562)

[Human articular cartilage 13](#_Toc102162563)

[Human cartilage samples 13](#_Toc102162564)

[*Ex vivo* imaging of human cartilage 13](#_Toc102162565)

[Supplemental Figures 14](#_Toc102162566)

[Fig. S1. DIT peptide HPLC Chromatograms 14](#_Toc102162567)

[Fig. S2. DIT peptide ^1^H-NMR Spectra 21](#_Toc102162568)

[Fig. S3. Concentration of iodine required for effective contrast enhancement of cartilage 34](#_Toc102162569)

[Fig. S4. Curves for DIT peptide radiocontrast agents 35](#_Toc102162570)

[Fig. S5*.* *Ex* vivo study of the minimum damage to murine cartilage that DIPIC can detect 36](#_Toc102162571)

[Fig. S6. Image analysis methodology 37](#_Toc102162572)

[References 38](#_Toc102162573)

# Methods

## Peptide chemistry

Peptide synthesis**.** Fmoc-amino acids and Wang resins were purchased from a combination of AGTC Bioproducts, Fluorochem, Chem-Impex and Novabiochem. Piperidine/DMF was acquired as a 20% (v/v) premix from AGTC Bioproducts. All reagents were used as received without further purification. Peptides were synthesised using the CEM Liberty Blue automated microwave peptide synthesiser. Fmoc deprotection of each amino acid was monitored using the internal UV detection software, with double-conventional couplings performed for residues that showed incomplete coupling. Synthesised peptides were purified using a Waters HPLC system with a Kinetex 5u EVO C18 100A column (150 x 21.2 mm) using a gradient of 5-50-5% or 10-90-10% solvent A (94 % H_2_O, 6 % acetonitrile, 0.01 M ammonium acetate, pH 6.0) and solvent B (19 % H_2_O, 81 % acetonitrile, 0.01 M ammonium acetate, pH 6.0) over 15 min with a flow rate of 20 ml/min. The DIT peptides were acquired as acetate salts with ≥ 95% purity by HPLC. Analytical QC runs were performed on a Kinetex 5u EVO C18 100A column (100 x 3.0 mm) in water and/or DMSO solvent. Lyophilisation was carried out on a Genevac EZ-2 Elite personal evaporator. LRMS data was obtained using a Waters SQ Detector 2 (ESI-MS). HRMS data was obtained on an Agilent 6530 accurate-mass Q-TOF LC/MS. Vortexing was performed on an IKA KS 260 Basic Shaker with centrifugation on an Eppendorf 5810R centrifuge. ^1^H-NMR spectra were acquired using a Bruker Ascend spectrometer at 400 MHz. Chemical shifts (δ) are referenced to residual solvent and reported in ppm, with coupling constants (*J*) quoted in Hz.

Peptide cleavage and purification**.** After completion of peptide synthesis, cleavage cocktail (95% trifluoroacetic acid: 2.5% triisopropylsilane: 2.5% H_2_O) was added to the Wang resin. The subsequent yellow-brown mixture was vortexed (6 h) and cooled (10 min, -26 °C). Ice-cold diethyl ether (50 ml) was added and the peptide left to precipitate overnight (-26 °C). The resultant precipitate was centrifuged (2906 *x* g, 4 °C, 10 min) and washed with ether (25 ml). The precipitate was dried under low vacuum (5 h) to deliver the peptide as a crude white to off-white powder. Peptides were used as is for initial testing, or further purified to ≥ 95% purity by HPLC for re-testing.

### Characterisation of DIT peptides

**W'Y'RGRL**

Purification by HPLC delivered an off-white solid (5.3 mg, 4%). **^1^H-NMR** (400 MHz, D_2_O): δ 7.56 (s, 2H, *DIT* ArH), 7.55-7.49 (m, 2H, *Trp* ArH), 7.30 (s, 1H, *Trp* HN-CH), 7.28-7.23 (m, 1H, *Trp* ArH), 7.19-7.13 (m, 1H, *Trp* ArH), 4.43 (dd, *J* = 8.6, 5.5 Hz, 1H, *DIT* Hα), 4.34 (t, *J* = 6.5 Hz, 1H, *Arg* Hα), 4.28 (t, *J* = 7.0 Hz, 1H, *Trp* Hα), 4.22-4.14 (m, 1H, *Leu* Hα), 4.06-3.97 (m, 2H, *Arg* Hα, *Gly* Hα), 3.84 (d, *J* = 17.1 Hz, 1H, *Gly* Hα), 3.36 (d, *J* = 7.1 Hz, 2H, *Trp* Hβ), 3.22-3.04 (m, 4H, *Arg* Hδ), 2.93-2.85 (m, 1H, *DIT* Hβ), 2.79 (dd, *J* = 13.9, 8.6 Hz, 1H, *DIT* Hβ), 1.87-1.69 (m, 2H, *Arg* 2Hβ), 1.69-1.54 (m, 7H, *Arg* 2Hβ, 2Hγ, *Leu* 2Hβ, Hγ), 1.53-1.43 (m, 2H, *Arg* Hγ), 0.90 (d, *J* = 6.1 Hz, 3H, *Leu* CH_3_), 0.85 (d, *J* = 6.1 Hz, 3H, *Leu* CH_3_) ppm; **ESI-MS** (m/z): 551.8 [M+2H]^2+^; **HRMS** (ESI-MS): calc. for C_40_H_58_I_2_N_13_O_8_ [M+H]^+^ 1102.2615 found 1102.2642.

**'Y'W'Y'RGRL**

HPLC purification gave a yellow solid (1.8 mg, 1%). **^1^H-NMR** (400 MHz, (CD_3_)_2_SO): δ 10.79 (s, 1H, *Trp* NH), 9.89 (s, 1H, *Tyr* OH), 8.30-8.07 (m, 3H, NH), 8.01-7.91 (m, 1H, NH), 7.48 (d, *J* = 7.9 Hz, 1H, ArH), 7.46-7.40 (m, 3H, ArH), 7.31 (d, *J* = 8.1 Hz, 1H, ArH), 7.24 (d, *J* = 7.3 Hz, 1H, ArH), 7.08-7.02 (m, 2H, ArH), 6.95 (d, *J* = 7.0 Hz, 1H, ArH), 6.55 (s, 1H, NH), 4.56-4.48 (m, 1H, Hα), 4.47-4.39 (m, 1H, Hα), 4.22-4.08 (m, 2H, Hα), 3.94-3.79 (m, 2H, Hα), 3.72-3.59 (m, 2H, Hα), 3.15-2.98 (m, 7H, CH_2_), 2.80-2.72 (m, 1H, CH_2_), 2.66-2.60 (m, 1H, CH_2_), 2.32-2.21 (m, 1H, CH_2_), 1.91-1.76 (m, 1H, CH_2_), 1.78-1.33 (m, 10H, CH_2_ *Leu* Hγ, 2Hβ), 0.87 (d, *J* = 2.6 Hz, 3H, CH_3_), 0.85 (d, *J* = 2.7 Hz, 3H, CH_3_) ppm; **ESI-MS** (m/z): 759.1 [M+2H]^2+^; **HRMS** (ESI-MS): calc. for C_49_H_65_I_4_N_14_O_10_ [M+H]^+^ 1517.1181 found 1517.1204.

**W'Y'KGKL**

Purification by HPLC delivered a white crystalline solid (23.5 mg, 19%). **^1^H-NMR** (400 MHz, D_2_O): δ 7.55-7.50 (m, 2H, *Trp* ArH), 7.50 (s, 2H, *DIT* ArH), 7.30 (s, 1H, *Trp* HN-CH), 7.28-7.23 (m, 1H, *Trp* ArH), 7.18-7.13 (m, 1H, *Trp* ArH), 4.38 (dd, *J* = 7.8, 5.1 Hz, 1H, *DIT* Hα), 4.25-4.17 (m, 3H, *Lys* 2Hα, *Trp* Hα), 4.08 (d, *J* = 17.2 Hz, 1H, *Gly* Hα), 3.98 (t, *J* = 7.2 Hz, 1H, *Leu* Hα), 3.80 (d, *J* = 17.1 Hz, 1H, *Gly* Hα), 3.41-3.29 (m, 2H, *Trp* Hβ), 2.99-2.91 (m, 4H, *Lys* Hε), 2.90-2.76 (m, 2H, *DIT* Hβ), 1.78-1.49 (m, 10H, *Lys* 4Hδ, 3Hβ, *Leu* Hγ, 2Hβ), 1.42-1.24 (m, 5H, *Lys* 4Hγ, Hβ), 0.90 (d, *J* = 6.1 Hz, 3H, *Leu* CH_3_), 0.86 (d, *J* = 6.1 Hz, 3H, *Leu* CH_3_) ppm; **ESI-MS** (m/z): 524.1 [M+2H]^2+^; **HRMS** (ESI-MS): calc. for C_40_H_58_I_2_N_9_O_8_ [M+H]^+^ 1046.2498 found 1046.2489.

**'Y'W'Y'KGKL**

Purification by HPLC gave an off-white solid (32.6 mg, 20%). **^1^H-NMR** (400 MHz, (CD_3_)_2_SO): δ 10.83 (s, 1H, *Trp* NH), 8.28-8.00 (m, 4H, NH), 7.93 (s, 1H, NH), 7.60 (s, 1H, NH), 7.48-7.36 (m, 5H, 4*DIT* ArH, *Trp* ArH), 7.28 (d, *J* = 8.0 Hz, 1H, *Trp* ArH), 7.05 (s, 1H, *Trp* HN-CH), 6.99 (t, *J* = 7.6 Hz, 1H, *Trp* ArH), 6.90 (t, *J* = 7.4 Hz, 1H, *Trp* ArH), 4.47 (s, 1H, *Trp* Hα), 4.42-4.32 (m, 1H, *DIT* Hα), 4.21-4.01 (m, 2H, *Lys* Hα), 3.98-3.89 (m, 2H, *DIT* Hα*,* *Leu* Hα), 3.79-3.59 (m, 3H, Gly 2Hα, *Trp* Hβ), 3.33-3.25 (m, 1H, *DIT* Hβ), 3.00 (s, 1H, *Trp* Hβ), 2.82-2.55 (m, 6H, *Lys* 2CH_2_, *DIT* 2Hβ), 2.31-2.19 (m, 1H, *DIT* Hβ), 1.71-1.35 (m, 11H, *Lys* 4CH_2_, *Leu* Hγ*,* 2Hβ), 1.34-1.10 (m, 4H, *Lys* CH_2_), 0.80 (d, *J* = 7.1 Hz, 6H, *Leu* CH_3_) ppm; **ESI-MS** (m/z): 731.5 [M+2H]^2+^; **HRMS** (ESI-MS): calc. for C_49_H_65_I_4_N_10_O_10_ [M+H]^+^ 1461.1064 found 1461.1040.

**'Y'WGKKL**

HPLC purification gave a yellow solid (22.8 mg, 19%). **^1^H-NMR** (400 MHz, D_2_O): δ 7.60 (d, *J* = 7.9 Hz, 1H, *Trp* ArH), 7.55 (s, 2H, *DIT* ArH), 7.49 (d, *J* = 8.1 Hz, 1H, *Trp* ArH), 7.27-7.21 (m, 1H, *Trp* ArH), 7.21 (s, 1H, *Trp* HN-CH), 7.18-7.13 (m, 1H, *Trp* ArH), 4.62 (t, *J* = 7.3 Hz, 1H, *Trp* Hα), 4.27-4.21 (m, 2H, *Lys* Hα), 4.21-4.16 (m, 1H, *Leu* Hα), 4.13 (dd, *J* = 7.8, 5.3 Hz, 1H, *DIT* Hα), 3.94 (d, *J* = 17.0 Hz, 1H, *Gly* Hα), 3.68 (d, *J* = 17.0 Hz, 1H, *Gly* Hα), 3.29-3.15 (m, 2H, *Trp* 2Hβ), 3.09 (dd, *J* = 14.3, 5.2 Hz, 1H, *DIT* Hβ), 2.99-2.87 (m, 5H, *DIT* Hβ, *Lys* 4Hε), 1.86-1.50 (m, 11H, *Lys* 4Hδ, 4Hβ, *Leu* 2Hβ, Hγ), 1.46-1.27 (m, 4H, *Lys* 4Hγ), 0.90 (d, *J* = 6.1 Hz, 3H, *Leu* CH_3_), 0.85 (d, *J* = 6.1 Hz, 3H, *Leu* CH_3_) ppm; **ESI-MS** (m/z): 524.1 [M+2H]^2+^; **HRMS** (ESI-MS): calc. for C_40_H_58_I_2_N_9_O_8_ [M+H]^+^ 1046.2498 found 1046.2503.

**K'Y'W'Y'KGKL**

Purification by HPLC gave an off-white solid (8.4 mg, 5%). **^1^H-NMR** (400 MHz, D_2_O): δ 7.59-7.38 (m, 4H, ArH), 7.38-7.25 (m, 2H, ArH), 7.25-7.13 (m, 2H, ArH), 7.13-7.00 (m, 1H, ArH), 4.69-4.53 (m, 2H, Hα), 4.52-4.32 (m, 1H, Hα), 4.32-4.16 (m, 2H, Hα), 4.16-3.97 (m, 2H, Hα), 3.97-3.75 (m, 2H, Hα), 3.26-3.05 (m, 2H, CH_2_), 3.05-2.84 (m, 5H, CH_2_), 2.85-2.60 (m, 5H, CH_2_), 1.80-1.45 (m, 14H, 6CH_2_, *Leu* Hγ, Hβ), 1.45-1.11 (m, 7H, 3CH_2_, *Leu* Hβ), 0.94-0.77 (m, 6H, *Leu* CH_3_) ppm; **ESI-MS** (m/z): 795.4 [M+2H]^2+^; **HRMS** (ESI-MS): calc. for C_55_H_77_I_4_N_12_O_11_ [M+H]^+^ 1589.2008 found 1589.1987.

**K'Y'KW'Y'KGKL**

HPLC purification afforded a white solid (8.5 mg, 4%): **^1^H-NMR** (400 MHz, D_2_O): δ 7.57-7.50 (m, 3H, *Trp* ArH, *DIT* 2ArH), 7.46 (d, *J* = 8.1 Hz, 1H, *Trp* ArH), 7.32-7.25 (m, 3H, *DIT* 2ArH, *Trp* HN-CH), 7.22 (t, *J* = 7.6 Hz, 1H, *Trp* ArH), 7.12 (t, *J* = 7.3 Hz, 1H, *Trp* ArH), 4.64-4.52 (m, 2H, *DIT* Hα), 4.43-4.33 (m, 1H, *Trp* Hα), 4.32-4.17 (m, 4H, *Lys* 3Hα, *Leu* Hα), 4.10 (d, *J* = 17.1 Hz, 1H, *Gly* Hα), 3.92 (t, *J* = 6.7 Hz, 1H, *Lys* Hα, br, 1H, *Gly* Hα), 3.36-3.26 (m, 1H, *DIT* Hβ), 3.26-3.18 (m, 1H, *DIT* Hβ), 3.00-2.82 (m, 9H, *Lys* 4CH_2_, *DIT* Hβ), 2.83-2.73 (m, 1H, *DIT* Hβ), 2.68-2.50 (m, 2H, *Trp* Hβ), 1.84-1.74 (m, 2H, *Lys* CH_2_), 1.73-1.50 (m, 15H, *Lys* 6CH_2_, *Leu* Hγ, 2Hβ), 1.43-1.05 (m, 10H, *Lys* 5CH_2_), 0.90 (d, *J* = 6.0 Hz, 3H, *Leu* CH_3_), 0.86 (d, *J* = 5.9 Hz, 3H, *Leu* CH_3_) ppm; **ESI-MS** (m/z): 859.2 [M+2H]^2+^; **HRMS** (ESI-MS): calc. for C_61_H_89_I_4_N_14_O_12_ [M+H]^+^ 1717.2958 found 1717.2936.

**K'Y'K'Y'W'Y'KGKL**

HPLC purification furnished a white solid (23.4 mg, 10%). **^1^H-NMR** (400 MHz, D_2_O, 333K): δ 7.89-7.74 (m, 6H, ArH), 7.73-7.64 (m, 2H, ArH), 7.55-7.45 (m, 2H, ArH), 7.44-7.36 (m, 1H, ArH), 5.10-4.86 (m, 2H, Hα), 4.63-4.50 (m, 3H, Hα), 4.42-4.30 (m, 2H, Hα), 4.08-4.00 (m, 1H, Hα), 3.62-3.48 (m, 3H, Hα), 3.39-3.20 (m, 10H, CH_2_), 3.09-3.02 (m, 2H, CH_2_), 2.21-1.87 (m, 21H, 10CH_2_, *Leu* Hγ), 1.80-1.50 (m, 10H, 4CH_2_, *Leu* 2Hβ), 1.28-1.16 (m, 6H, *Leu* CH_3_). **ESI-MS** (m/z): 1066.6 [M+2H]^2+^; **HRMS** (ESI-MS): calc. for C_70_H_96_I_6_N_15_O_14_ [M+H]^+^ 2132.1524 found 2132.1545.

**'Y'-*AEEA*-WYKGKL**

HPLC purification yielded an off-white solid (40.4 mg, 26%). **^1^H-NMR** (400 MHz, D_2_O): δ 7.51-7.49 (m, 1H, *Trp* ArH), 7.48 (s, 2H, *DIT* ArH), 7.44 (d, *J* = 8.1 Hz, 1H, *Trp* ArH), 7.20-7.14 (m, 1H, *Trp* ArH), 7.14 (s, 1H, *Trp* HN-CH), 7.08 (t, *J* = 7.4 Hz, 1H, *Trp* ArH), 6.90 (d, *J* = 8.4 Hz, 2H, *Tyr* ArH), 6.72 (d, *J* = 8.4 Hz, 2H, *Tyr* ArH), 4.64 (t, *J* = 6.7 Hz, 1H, *Trp* Hα), 4.46 (t, *J* = 7.1 Hz, 1H, *Tyr* Hα), 4.30 (t, *J* = 7.2 Hz, 1H, *Lys* Hα), 4.20 (t, *J* = 7.1 Hz, 1H, *Leu* Hα), 4.14 (dd, *J* = 8.3, 6.1 Hz, 1H, *Lys* Hα), 3.98 (dd, *J* = 9.0, 6.1 Hz, 1H, *DIT* Hα), 3.95-3.83 (m, 4H, *AEEA* CH_2_, Gly 2Hα), 3.51-3.28 (m, 6H, *AEEA* CH_2_), 3.23-3.03 (m, 4H, *AEEA* CH_2_, *Trp* 2Hβ), 3.01-2.88 (m, 5H, *DIT* Hβ, *Lys* 2CH_2_), 2.87-2.72 (m, 3H, *Tyr* 2Hβ, *DIT* Hβ), 1.87-1.69 (m, 2H, *Lys* CH_2_), 1.68-1.52 (m, 9H, *Lys* 3CH_2_, *Leu* Hγ, 2Hβ), 1.45-1.32 (m, 2H, *Lys* CH_2_), 1.33-1.21 (m, 2H, *Lys* CH_2_), 0.86 (d, *J* = 5.9 Hz, 3H, *Leu* CH_3_), 0.82 (d, *J* = 5.7 Hz, 3H, *Leu* CH_3_) ppm; **ESI-MS** (m/z): 678.2 [M+2H]^2+^; **HRMS** (ESI-MS): calc. for C_55_H_78_I_2_N_11_O_13_ [M+H]^+^ 1354.3870 found 1354.3870.

**'Y''Y'-*AEEA*-WYKGKL**

HPLC purification furnished a white solid (9.2 mg, 5%). **^1^H-NMR** (400 MHz, (CD_3_)_2_SO): δ 11.04 (s, 1H *Trp* NH), 8.33 (d, *J* = 7.9 Hz, 1H, NH), 8.14 (d, *J* = 7.2 Hz, 1H, NH), 8.08-7.87 (m, 4H, NH), 7.67 (d, *J* = 7.2 Hz, 1H, NH), 7.57 (d, *J* = 7.8 Hz, 1H, NH), 7.53 (d, *J* = 7.9 Hz, 1H, *Trp* ArH), 7.49 (s, 2H, *DIT* ArH), 7.36-7.32 (m, 3H, *Trp* ArH, *DIT* 2ArH), 7.08 (s, 1H, *Trp* HN-CH), 7.06-7.01 (m, 1H, *Trp* ArH), 7.00 (d, *J* = 8.5 Hz, 2H, *Tyr* ArH), 6.95 (t, *J* = 7.4 Hz, 1H, *Trp* ArH), 6.63 (d, *J* = 8.4 Hz, 2H, *Tyr* ArH), 4.56 (dd, *J* = 7.5 Hz, 1H, *Trp* Hα), 4.47 (dd, *J* = 7.4 Hz, 1H, *Tyr* Hα), 4.40-4.31 (m, 1H, *DIT* Hα), 4.30-4.17 (m, 2H, *Lys* Hα), 4.00-3.91 (m, 1H, *Leu* Hα), 3.80 (d, *J* = 15.5 Hz, 1H, *Gly* Hα), 3.75-3.65 (m, 2H, *Gly* Hα, *DIT* Hα), 3.39-3.33 (m, 4H, *AEEA* CH_2_, *DIT* 2Hβ), 3.32-3.16 (m, 6H, *AEEA* CH_2_), 3.13-3.01 (m, 4H, *AEEA* CH_2_, *Trp* 2Hβ), 2.98-2.87 (m, 1H, *Tyr* Hβ), 2.80-2.61 (m, 7H, *Lys* 2CH_2_, *DIT* 2Hβ, *Tyr* Hβ), 1.77-1.39 (m, 11H, *Lys* 4CH_2_, *Leu* Hγ, 2Hβ), 1.39-1.21 (m, 4H, *Lys* CH_2_), 0.86 (d, *J* = 6.6 Hz, 3H, *Leu* CH_3_), 0.83 (d, *J* = 6.6 Hz, 3H, *Leu* CH_3_) ppm; **ESI-MS** (m/z): 885.9 [M+2H]^2+^; **HRMS** (ESI-MS): calc. for C_64_H_85_I_4_N_12_O_15_ [M+H]^+^ 1769.2431 found 1769.2459.

**WYKGKL-*AEEA*-'Y'G**

HPLC purification furnished an off-white solid (29.1 mg, 18%). **^1^H-NMR** (400 MHz, D_2_O): δ 7.51 (d, *J* = 7.8 Hz, 1H, *Trp* ArH), 7.45 (s, 1H, *Trp* ArH), 7.43 (s, 2H, *DIT* ArH), 7.22 (s, 1H, *Trp* HN-CH), 7.17 (t, *J* = 7.6 Hz, 1H, *Trp* ArH), 7.07 (t, *J* = 7.5 Hz, 1H, *Trp* ArH), 6.93 (d, *J* = 8.1 Hz, 2H, *Tyr* ArH), 6.73 (d, *J* = 8.0 Hz, 2H, *Tyr* ArH), 4.63–4.57 (m, 1H, *DIT* Hα), 4.47 (t, *J* = 7.2 Hz, 1H, *Tyr* Hα), 4.34–4.26 (m, 2H, *Lys* Hα), 4.20 (t, *J* = 6.7 Hz, 1H, *Trp* Hα), 4.15–4.09 (m, 1H, *Leu* Hα), 4.02–3.69 (m, 6H, *AEEA* CH_2_, *Gly* 4Hα), 3.55–3.20 (m, 10H, *AEEA* 4CH_2_, *Trp* 2Hβ), 2.99–2.90 (m, 3H, *Lys* CH_2_, *DIT* Hβ), 2.91–2.80 (m, 4H, *Lys* CH_2_, *Tyr* 2Hβ), 2.69–2.58 (m, 1H, *DIT* Hβ), 1.83–1.67 (m, 3H, *Leu* Hβ, *Lys* CH_2_), 1.66–1.46 (m, 8H, *Lys* 3CH_2_, *Leu* Hβ, *Leu* Hγ), 1.42–1.29 (m, 2H, *Lys* CH_2_), 1.30–1.13 (m, 2H, *Lys* CH_2_), 0.80 (d, *J* = 5.4 Hz, 3H, *Leu* CH_3_), 0.76 (d, *J* = 5.3 Hz, 3H, *Leu* CH_3_) ppm; **ESI-MS** (m/z): 706.9 [M+2H]^2+^; **HRMS** (ESI-MS): calc. for C_57_H_81_I_2_N_12_O_14_ [M+H]^+^ 1411.4085 found 1411.4063.

**W'Y'KGKL-*AEEA*-'Y'G**

HPLC purification delivered an off-white solid (5.9 mg, 4%). **^1^H-NMR** (400 MHz, (CD_3_)_2_SO): δ 10.90 (s, 1H, *Trp* NH), 8.43-8.37 (m, 1H, NH), 8.30 (s, 1H, NH), 8.27-8.20 (m, 1H, NH), 8.10 (s, 1H, NH), 7.99-7.93 (m, 1H, NH), 7.89-7.81 (m, 3H, NH), 7.56 (d, *J* = 8.1 Hz, 1H, *Trp* ArH), 7.47 (s, 2H, *DIT* ArH), 7.37 (s, 2H, *DIT* ArH), 7.34 (d, *J* = 8.1 Hz, 1H, *Trp* ArH), 7.20-7.16 (m, 1H, *Trp* HN-CH), 7.06 (t, *J* = 7.3 Hz, 1H, *Trp* ArH), 6.97 (t, *J* = 7.2 Hz, 1H, *Trp* ArH), 4.57-4.44 (m, 2H, *DIT* Hα), 4.28-4.18 (m, 3H, *Trp* Hα, *Lys* Hα, *Leu* Hα), 4.17-4.07 (m, 1H, *Lys* Hα), 3.88 (d, *J* = 15.5 Hz, 2H, *Gly* Hα), 3.81 (d, *J* = 15.5 Hz, 2H, *Gly* Hα), 3.57-3.46 (m, 13H, *AEEA* 5CH_2_, *DIT* Hβ, *Trp* 2Hβ), 3.10-3.06 (m, 1H, *DIT* Hβ), 2.86-2.78 (m, 1H, *DIT* Hβ), 2.78-2.59 (m, 5H, *Lys* 2CH_2_, *DIT* Hβ), 1.68-1.40 (m, 11H, *Lys* 4CH_2_, *Leu* 2Hβ, Hγ), 1.30-1.15 (m, 4H, *Lys* CH_2_), 0.83 (d, *J* = 6.5 Hz, 3H, *Leu* CH_3_), 0.78 (d, *J* = 6.4 Hz, 3H, *Leu* CH_3_) ppm; **ESI-MS** (m/z): 832.2 [M+2H]^2+^; **HRMS** (ESI-MS): calc. for C_57_H_79_I_4_N_12_O_14_ [M+H]^+^ 1663.2018 found 1663.2043.

**K'Y'K'Y'-*AEEA*-WYKGKL**

HPLC purification delivered a white solid (6.2 mg, 3%). **^1^H-NMR** (400 MHz, D_2_O): δ 7.46 (d, *J* = 7.9 Hz, 1H, *Trp* NH), 7.44-7.39 (m, 3H, *DIT* 2ArH, *Trp* ArH), 7.21 (s, 1H, *Trp* HN-CH), 7.17 (t, *J* = 7.6 Hz, 1H, *Trp* ArH), 7.12 (s, 2H, *DIT* ArH), 7.04 (t, *J* = 7.3 Hz, 1H, *Trp* ArH), 6.89 (d, *J* = 8.3 Hz, 2H, *Tyr* ArH), 6.65 (d, *J* = 8.5 Hz, 2H, *Tyr* ArH), 4.93 (t, *J* = 6.3 Hz, 1H, *Trp* Hα), 4.64 (dd, *J* = 8.1, 5.9 Hz, 1H, *DIT* Hα), 4.44 (t, *J* = 7.7 Hz, 1H, *DIT* Hα), 4.39-4.30 (m, 2H, *Lys* Hα), 4.28-4.22 (m, 1H, *Lys* Hα), 4.20-4.15 (m, 1H, *Leu* Hα), 4.03-3.94 (m, 3H, *Tyr* Hα, *Gly* 2Hα), 3.93-3.88 (m, 1H, *Lys* Hα), 3.52-3.35 (m, 4H, *AEEA* CH_2_), 3.34-3.11 (m, 6H, *AEEA* 2CH_2_, *Trp* 2Hβ), 3.01-2.82 (m, 12H, *Tyr* 2Hβ, *AEEA* CH_2_, *Lys* 4CH_2_), 2.81-2.74 (m, 2H, *DIT* Hβ), 2.72-2.64 (m, 1H, *DIT* Hβ), 2.39-2.30 (m, 1H, *DIT* Hβ), 1.86-1.74 (m, 4H, *Lys* CH_2_), 1.73-1.50 (m, 15H, *Lys* 6CH_2_, *Leu* 2Hβ, Hγ), 1.45-1.35 (m, 4H, *Lys* CH_2_), 1.36-1.17 (m, 4H, *Lys* CH_2_), 0.87 (d, *J* = 5.8 Hz, 3H, *Leu* CH_3_), 0.84 (d, *J* = 5.8 Hz, 3H, *Leu* CH_3_) ppm; **ESI-MS** (m/z): 1013.2 [M+2H]^2+^; **HRMS** (ESI-MS): calc. for C_76_H_109_I_4_N_16_O_17_ [M+H]^+^ 2025.4330 found 2025.4327.

## Micro-CT imaging

General information**.** Scanning was performed on a Quantum FX micro-CT instrument (PerkinElmer, Waltham, Massachusetts, USA) at a spatial resolution of 10 μm/pixel (200 μA, 90 kV, 3 min acquisition time). Scans were reconstructed to generate cross-sectional images using the manufacturer’s built-in software (version 2.3, PerkinElmer, Waltham, Massachusetts, USA) and subsequently resliced for analysis using ImageJ (National Institutes of Health, Bethesda, Maryland, USA).

Iodine calibration curve**.** To determine the variation of grey level intensity with iodine concentration, solutions of diatrizoic acid (Alfa Aesar, Heysham, UK) were made up at 50.00, 25.00, 12.50, 6.25, 3.12, 1.56, 0.78 and 0.39 mg iodine/ml concentrations. To derive the calibration curve, the solutions were imaged by micro-CT and the average grey level intensity within a cylindrical volume of interest (VOI) was measured for each concentration using ImageJ (National Institutes of Health, Bethesda, Maryland, USA). From this curve, grey level intensity measurements of cortical bone (3250) and saline solution (285) enabled the upper and lower limits of radiocontrast agent concentrations to be defined (Fig. S4*A*). While a concentration of 50 mg iodine/ml provided a level of intensity that could not be differentiated from cortical bone (upper grey area in Fig. S4*A*), 1.56 mg iodine/ml gave a similar intensity to saline (lower grey area in Fig. S4*A*). A concentration of 20 mg iodine/ml provided contrast that clearly differentiated the radiocontrast agent (and thus the articular cartilage) from adjacent tissues and was therefore used in all subsequent experiments.

## Animal study

## Murine articular cartilage

Fluorescence immunohistochemistry for localisation of Ac-WYRGRL-DOTAM-Cy5.5**.** Fixed cryosections of murine tibiae (10 µm) 24 h after intra-articular injection of Ac-WYRGRL-DOTAM-Cy5.5 or Ac-YRLGRW-DOTAM-Cy5.5 [1] were blocked with 10% goat serum, stained with rat anti-heparan sulfate proteoglycan antibody (A7L6, Abcam), washed three times with PBS-Tween 0.05% (v/v), labelled with Alexa Fluor 488 goat anti-rat IgG (H+L, Abcam) and washed three times with PBS-Tween 0.05% (v/v) before imaging. Cryosections were viewed under a Nikon TE2000-U Ultraview confocal microscope (PerkinElmer, Seer Green, UK) using a 488 nm excitation laser with 525 nm emission filter to visualise the Alexa Fluor 488 label (green false colour), and a 640 nm excitation laser with 700 nm emission filter to visualise the Cy5.5 label (red false colour).

Calculation of *ex vivo* DIT peptide half-life**.** To determine the half-life of each peptide, a VOI (500 μm) that extended across the anterior-posterior axis was manually placed in the tibial articular cartilage and the average grey level intensity within it measured. After washing in saline, the amount (percentage) of peptide remaining in cartilage at time *t* was defined as the ratio between the average grey level intensity at time *t* and at equilibrium using equation 1:

$${Remaining peptide}_{t}\left( \% \right)=\frac{{grey level intensity}_{t}}{{grey level intensity}_{equilibrium}}\times100 (1)$$

Where *t* = the number of hours in saline. These quantifications provided the percentage decrease in contrast over time and were used to determine the half-life of each sequence by fitting the data to a model of one-phase decay using GraphPad Prism 7 (San Diego, California, USA).

X-ray fluorescence imaging of *ex vivo* murine articular cartilage**.** A murine tibia was incubated with **ˈYˈ**-*AEEA*-WYKGKL until saturation was achieved (2 h). This was followed by washing with saline (500 µl) for 30 min. The tibia was then transferred into optical cutting temperature compound (Sakura Finetek, Alphen aan den Rijn, Netherlands) and flash frozen in liquid nitrogen. Slices (10 µm) were then cryo-sectioned and dried on an X-ray transparent silicon nitride window (Silson Ltd, Southam, UK). X-ray fluorescence (XRF) experiments were carried out at the Nanoprobe beamline of Diamond Light Source (Didcot, UK) using a photon energy of 6.0 keV. A pair of pre-shaped Kirkpatrick-Baez mirrors were used to focus the X-ray beam to a size of 50 x 65 nm, and a 4-element XRF detector (RaySpec, High Wycombe, UK) positioned in backscatter geometry 17 mm from the sample was used to collect its X-ray emission. The silicon nitride window was mounted on a beamline-provided custom holder which inserted into the sample stage. Data collection was performed by raster-scanning the sample across the beam in a continuous fashion at constant velocity. As the beam size remained constant throughout the experiment, the resolution reported for the resulting images corresponded to the distance between rows in the scan (for the Y direction of the image), and to the distance over which the XRF signal was collected into a single spectrum (for the X direction of the image). As a result, the X-ray dose was delivered to constant-width lines across the selected region of interest, which cover the whole sample only at native beamline resolution. PyMCA software [2] was used to analyse the collected spectra. All relevant effects were considered in the fitting routine, including atmospheric absorption, secondary excitation and detector escape peaks.

*Ex vivo* imaging with DIPIC, PTA or iopamidol**.**  Mice were culled through inhalation of CO_2_ and their knee joints harvested and split under a dissection microscope. Tibiae (*n* = 3) were finely dissected to remove soft tissue and incubated overnight either in DIT peptide or iopamidol (Santa Cruz Biotechnology, Dallas, Texas, USA) at 20 mg iodine/ml (20 μl) or 1% PTA (Sigma) in 70% ethanol [3]. To determine the contrast achieved in the articular cartilage at equilibrium, proximal tibiae were imaged by micro-CT while incubated in each contrast agent. Equilibrium was defined as the time of incubation required to obtain two consecutive scans of articular cartilage without changes in its average absorption, a readout that indicated that the maximum amount of peptide bound to cartilage was reached. Following imaging, tibiae were washed in saline and imaged in fresh saline until the articular cartilage could no longer be detected.

Generation of colour-coded absorption maps**.** To calibrate micro-CT scans in Hounsfield units (HU), air and water were scanned and reconstructed. The average grey level intensity within a cylindrical VOI was obtained from image histograms in ImageJ (air = -832.85, water = 152.54). Based on knowledge of the average grey level intensity and the corresponding HU (air = -1000, water = 0), the following linear transformation was derived (equation 2):

$$HU=1.01\times Grey level intensity-154.80 (2)$$

Using this calibration, the grey level intensity of each pixel was linearly transformed into X-ray absorption (HU) for each micro-CT image dataset using Matlab (R2014b, MathWorks, Natick, Massachusetts, USA). To enhance the difference between X-ray absorption of each tissue, colour-coded maps, also expressed in HU, were generated in Matlab by assigning a look up table (LUT) of 256 colours to each image dataset.

Surgical induction of OA through destabilisation of the medial meniscus (DMM)**.** Four groups of 10-week-old male C57Bl/6 mice (*n* = 6 per group) underwent surgical destabilisation of the medial meniscus in the left knee joint, a procedure that involved surgical transection of the medial menisco-tibial ligament [4], leading to joint instability and cartilage loss. The contralateral limb was not operated on and served as an internal control (contralateral). Groups of animals were subsequently culled at 2, 4, 8 and 12 weeks post-surgery.

*In vivo* imaging in DMM mice using DIPIC**.** Mice were anaesthetised by inhalation of isoflurane (3% in medical oxygen for induction and 2% in medical oxygen for maintenance) and intra-articularly injected with DIT peptide radiocontrast agent in the right knee joint (20 μl, 20 mg iodine/ml) and saline solution in the left knee joint (20 μl). Joints were immediately imaged by micro-CT and animals were sacrificed by cervical dislocation thereafter.

*Ex vivo* quantitative imaging of DMM mice using PTA and DIPIC**.** Knee joints from osteoarthritic mice were harvested, split under a dissection microscope and tibiae finely dissected. Upon overnight incubation in the DIT peptide **ˈYˈ**-*AEEA*-WYKGKL, micro-CT imaging was performed at a spatial resolution of 10 µm/pixel. Samples were washed in saline thereafter and subsequently incubated in 1% PTA in 70% ethanol for PTA-CT imaging [3] using the same settings as for DIPIC. To quantify articular cartilage thickness using both types of probes, an automated method based on segmentation and mapping of volumes-of-interest (VOIs) was applied. For PTA-CT, the methodology utilised has been fully described elsewhere.[3] For DIPIC, although VOI mapping and quantification were accomplished using the same approach, a distinct segmentation technique was developed. Colour-coded maps, which more evidently enhanced the boundary between bone and cartilage than grey level intensities, were used to split tissues with high absorption (red channel, corresponding to X-ray absorptions above 2500 HU and mostly comprising cortical bone) from tissues with lower absorption (green and blue channels, corresponding to X-ray absorptions ranging from 800 to 2250 HU and comprising not only articular cartilage but also trabecular bone). The red channel was used to create a mask to remove any bony structures from the green and blue channels, based on binarization and morphological operations, thus resulting in segmentation of the cartilage layer. Subsequent VOI mapping and quantification were completed, and measurements obtained by DIPIC and PTA-CT compared by parametric correlations.

Histopathology of DMM mice**.** Tibiae were decalcified in 10% EDTA for 5 weeks, dehydrated, cleared, and embedded in paraffin. Coronal sections were obtained across the samples at a regular spacing (80 μm) in a total of 12 levels and stained using haematoxylin and eosin/safranin-O. Histological images were acquired by light microscopy to inspect for the presence of cartilage lesions, which were semi-quantitatively graded using the histological scoring system for murine OA defined by Osteoarthritis Research Society International (OARSI) [5]. For validation of thickness measurements, manual contouring of articular cartilage followed by thickness measurements using ImageJ (National Institutes of Health, Bethesda, Maryland, USA) was applied.

Statistical analysis**.** All statistical analysis was conducted using GraphPad Prism 7 (San Diego, California, USA).

## Human articular cartilage

Human cartilage samples**.** Human osteochondral plugs (*n* = 2) were incubated in **ʹYʹ**-*AEEA*-WYKGKL for 48 h and micro-CT imaging performed at a spatial resolution of 10 µm/pixel (30 min, 1 h, 2 h, 3 h, 4 h, 6 h, 8 h, 30 h and 48 h) to follow the diffusion of DIT peptide into tissue. Plugs were subsequently washed in saline up to 120 h and micro-CT imaging performed at 30 min, 1 h, 2 h, 3 h, 4 h, 6 h, 24 h, 48 h and 120 h to track the dynamics of DIT peptide washout.

*Ex vivo* imaging of human cartilage. Osteochondral plugs from tibiae of patients undergoing knee arthroplasty were collected, washed and frozen. Upon thawing, samples were placed in PCR tubes (0.25 ml, Thermo Fisher Scientific, Loughborough, UK) and DIT peptide **ʹYʹ**-*AEEA*-WYKGK (200 µl, 20 mg iodine/ml) was added to each osteochondral plug (*n* = 2). Micro-CT imaging was performed at a spatial resolution of 10 µm/pixel to follow the diffusion of DIT peptide into tissue (30 min, 1 h, 2 h, 3 h, 4 h, 6 h, 8 h, 30 h and 48 h of incubation in the probe). Plugs were subsequently washed in saline and placed in PCR tubes containing fresh saline up to 120 h. Micro-CT imaging was performed upon 30 min, 1 h, 2 h, 3 h, 4 h, 6 h, 24 h, 48 h and 120 h in saline to track the dynamics of DIT peptide clearance from the tissue.

# Supplemental Figures

### Fig. S1. DIT peptide HPLC Chromatograms

**W'Y'RGRL**

**
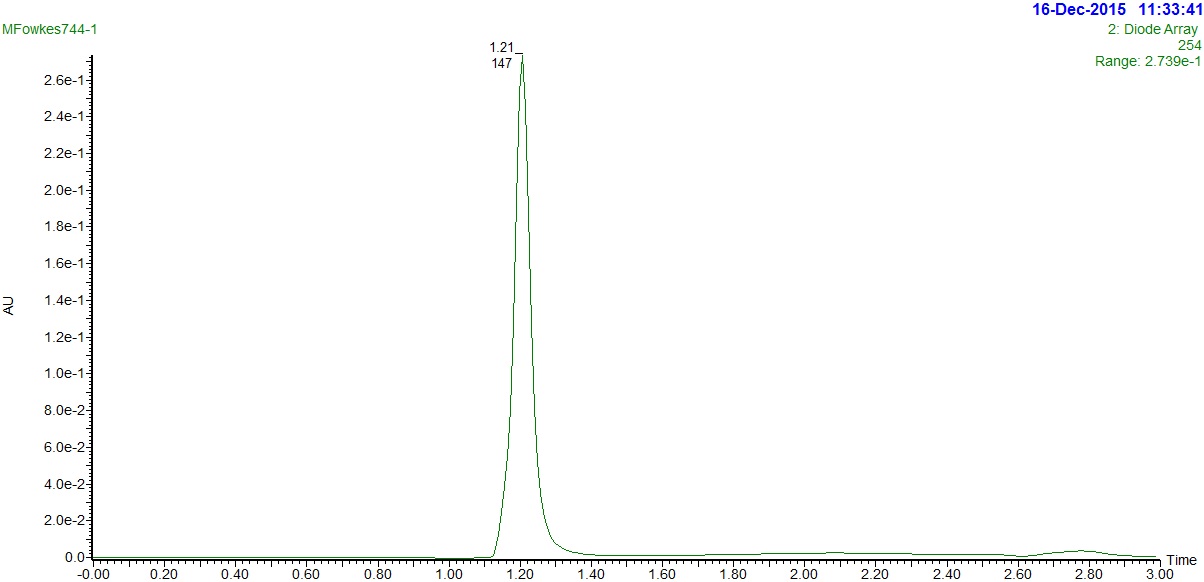
**

**'Y'W'Y'RGRL**

**
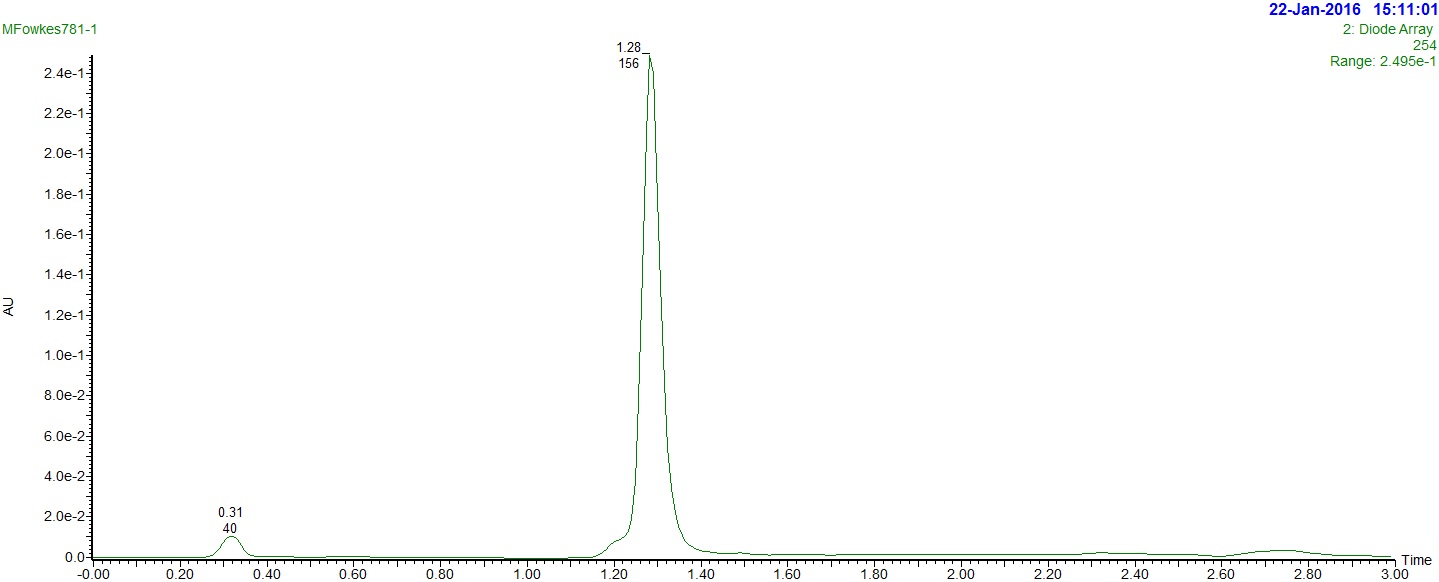
**

**W'Y'KGKL**


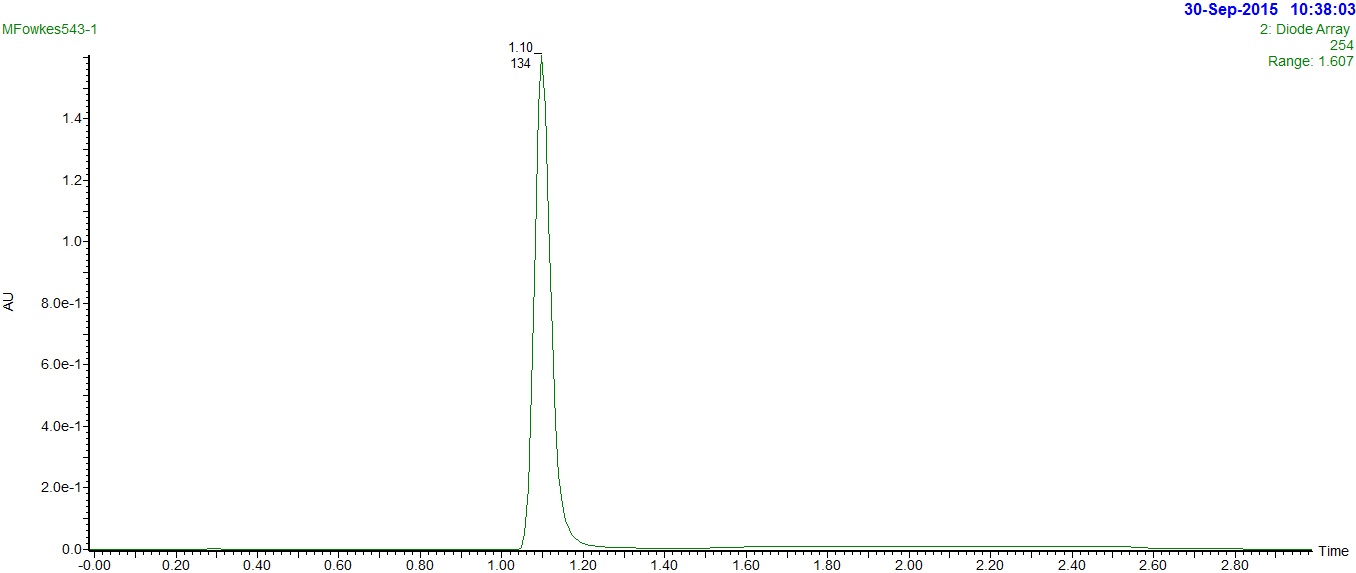


**'Y'W'Y'KGKL**


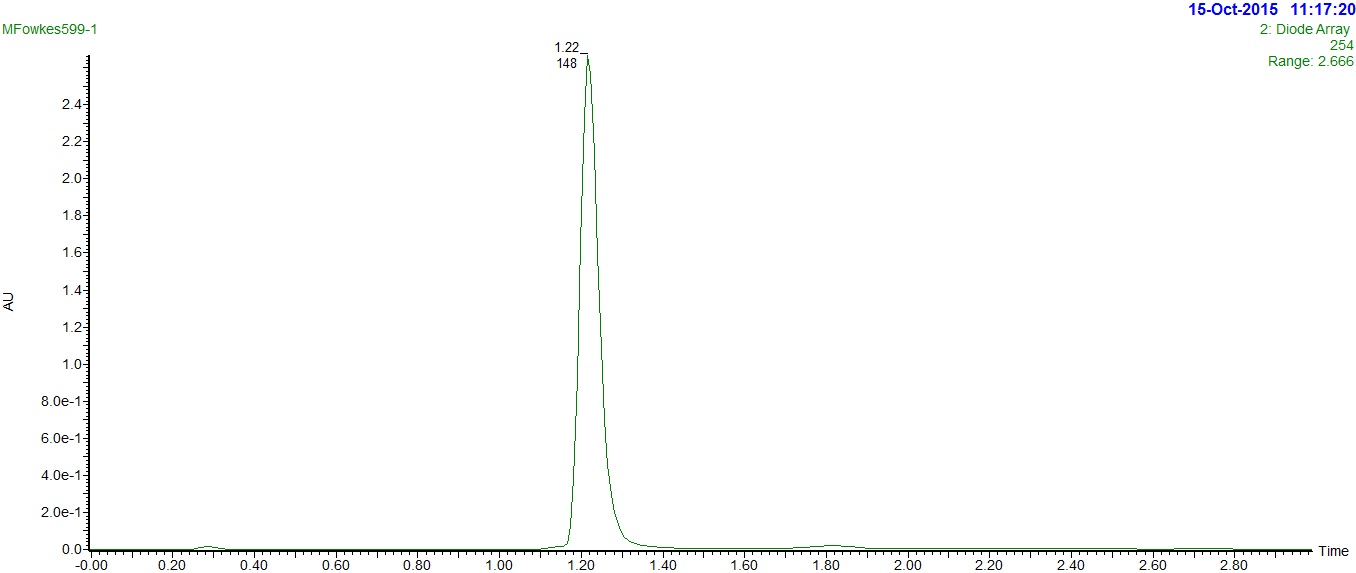


**'Y'WGKKL**

**
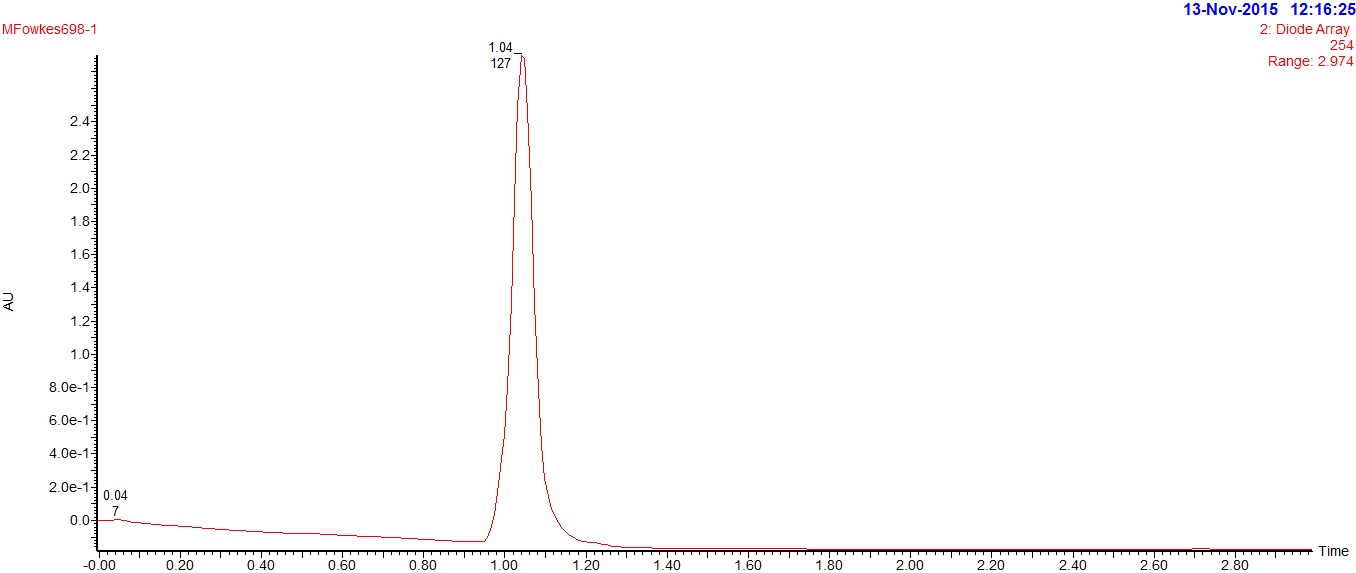
**

**K'Y'W'Y'KGKL**

**
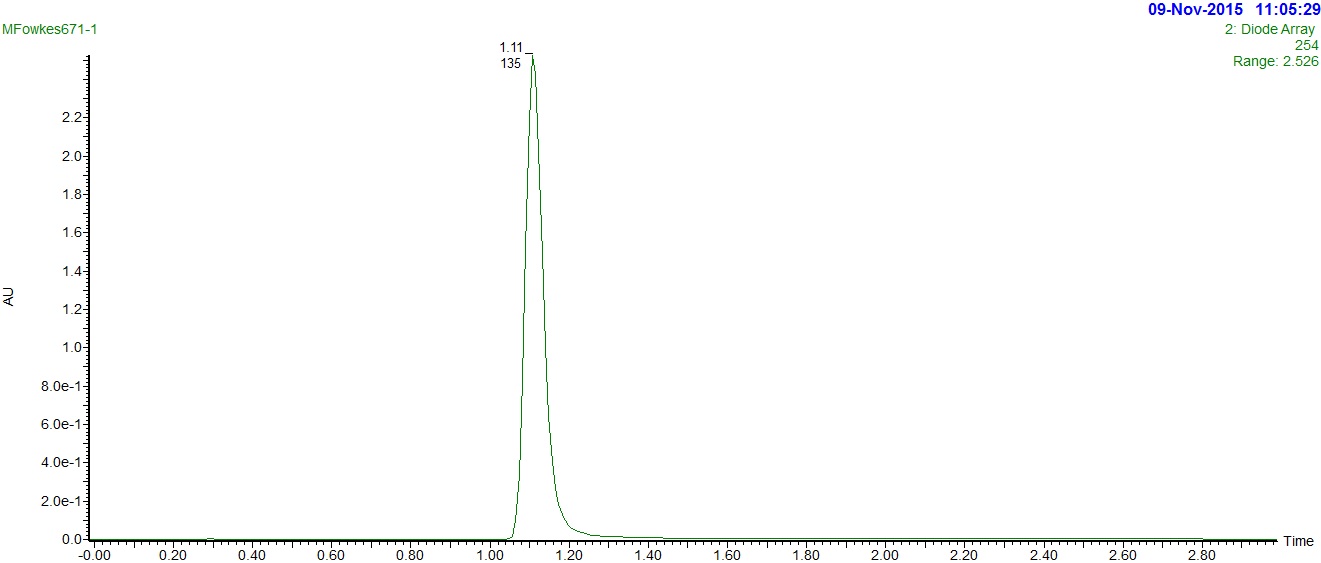
**

**K'Y'KW'Y'KGKL**

**
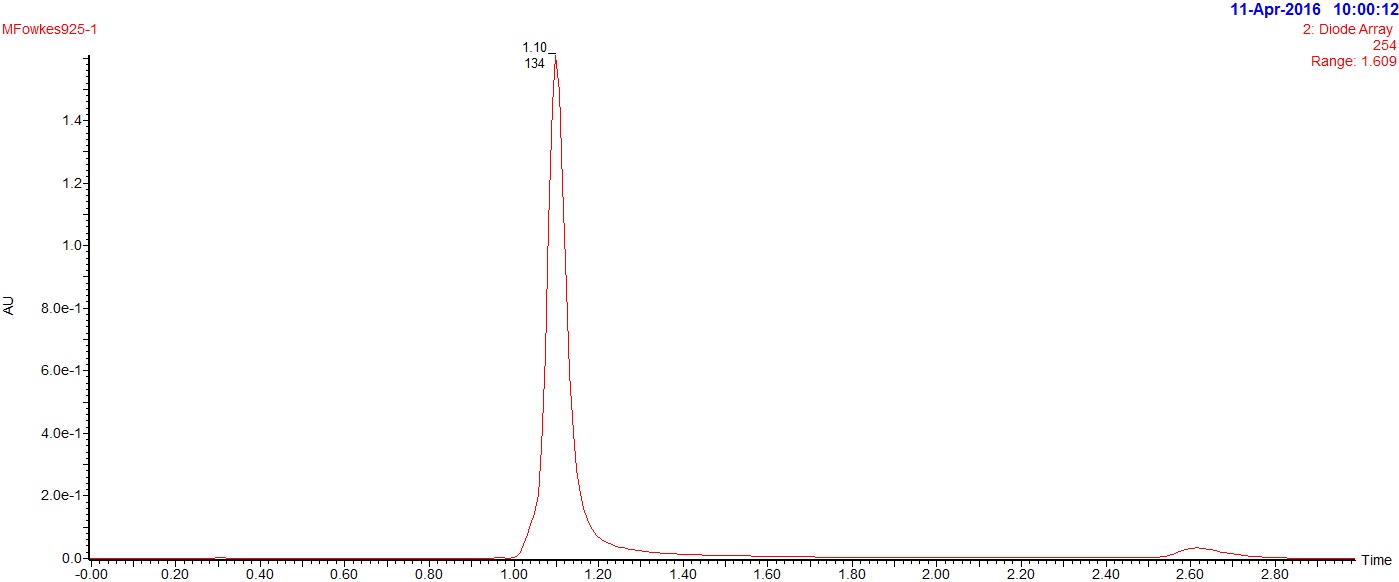
**

**K'Y'K'Y'W'Y'KGKL**

**
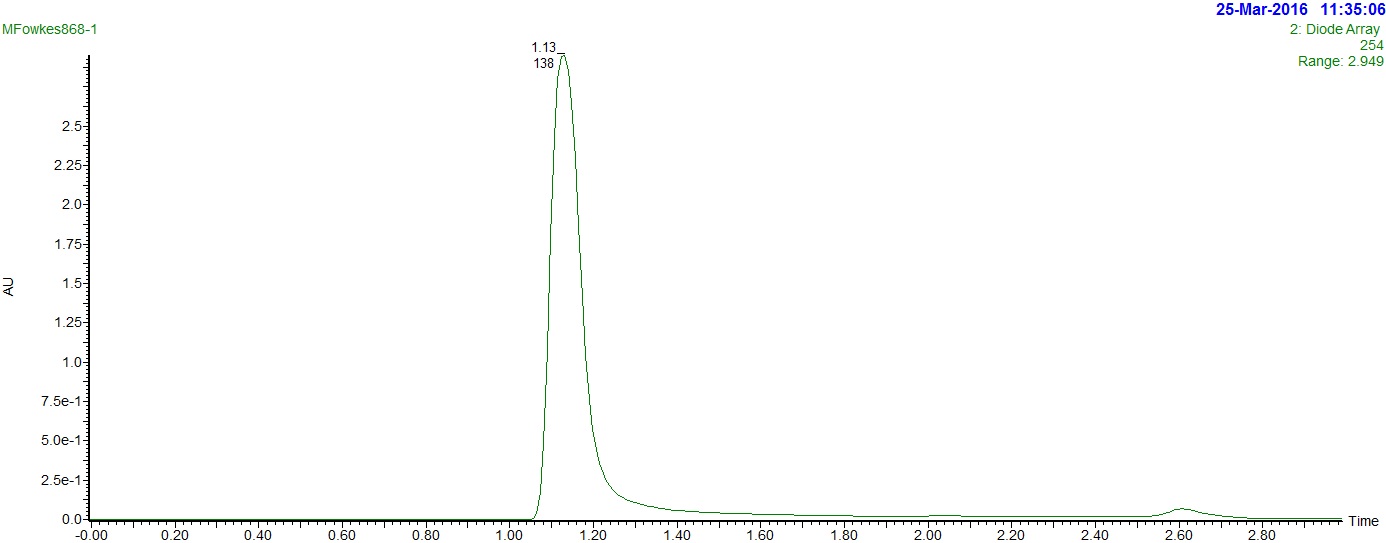
**

**'Y'-*AEEA*-WYKGKL**

**
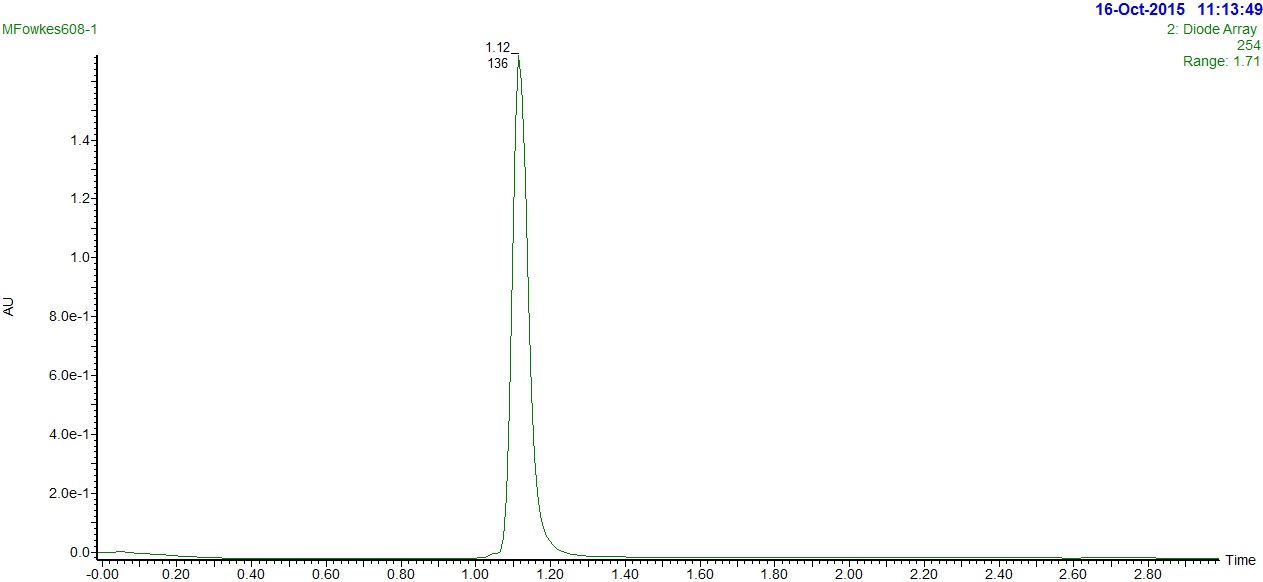
**

**'Y''Y'-*AEEA*-WYKGKL**

**
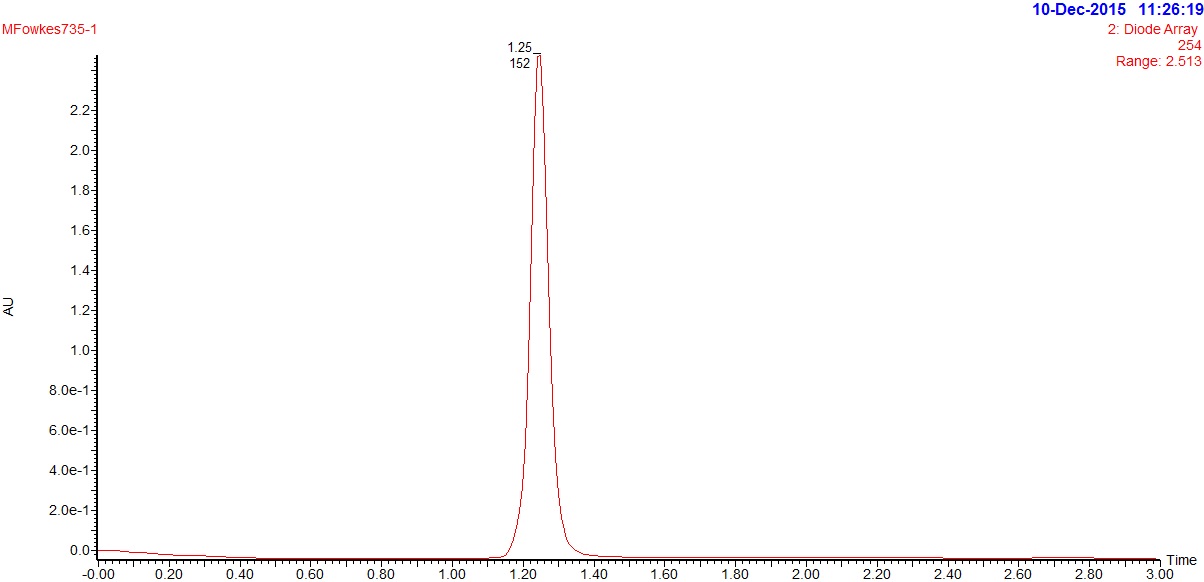
**

**WYKGKL-*AEEA*-'Y'G**

**
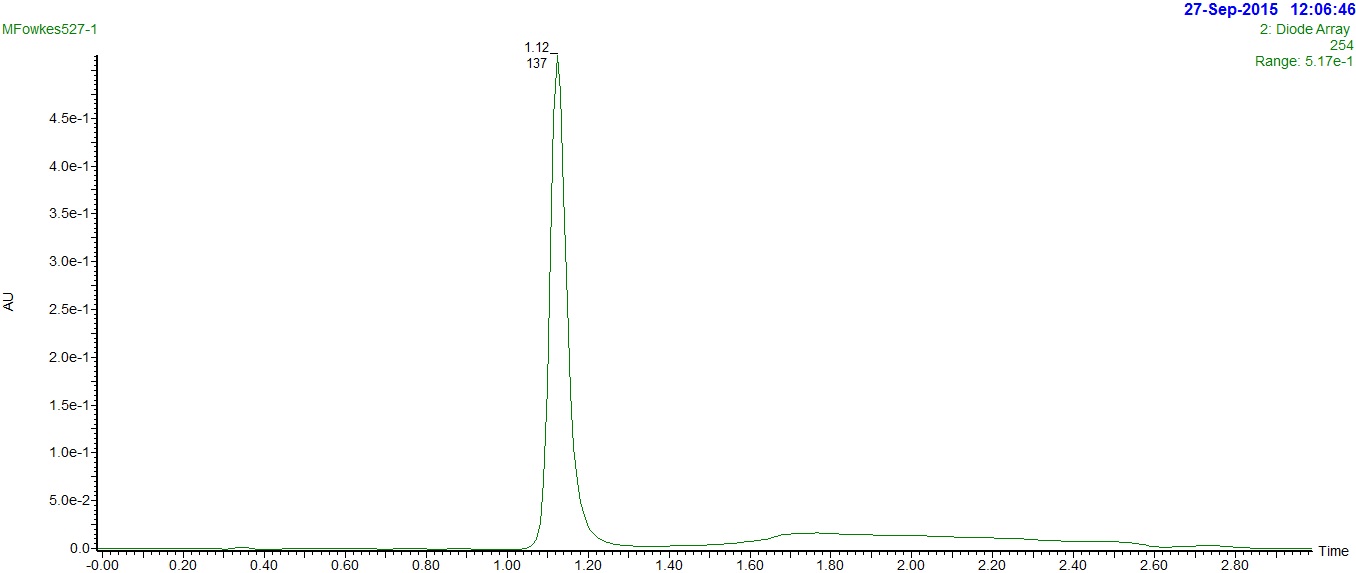
**

**W'Y'KGKL-*AEEA*-'Y'G**

**
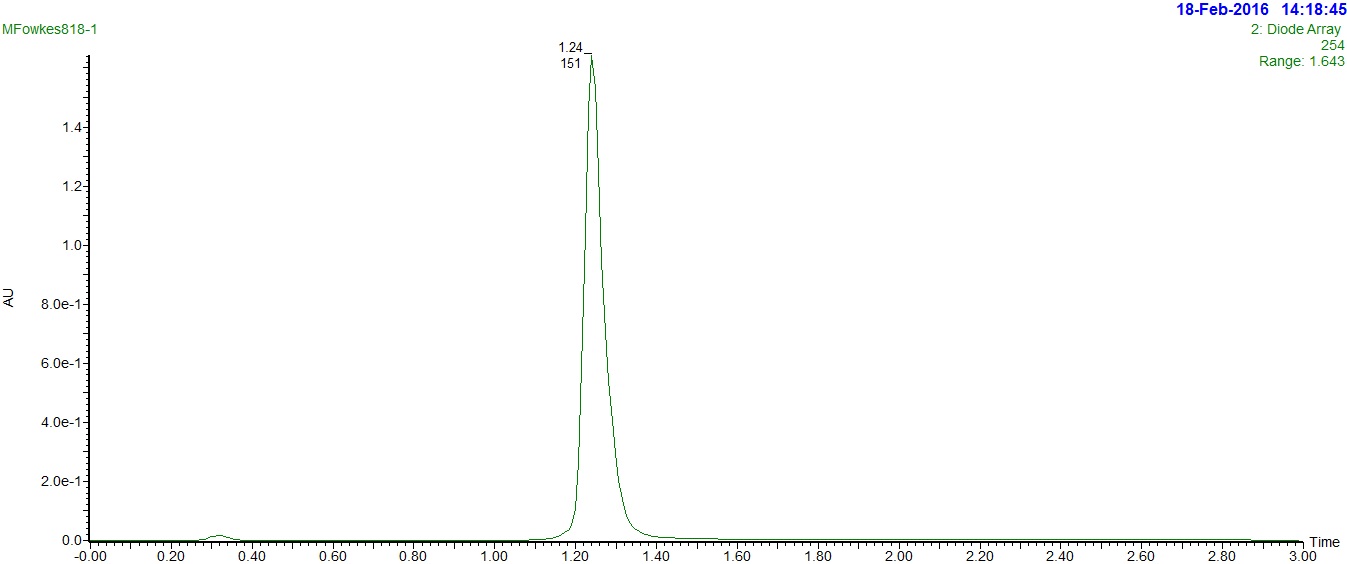
**

**K'Y'K'Y'-*AEEA*-WYKGKL**

**
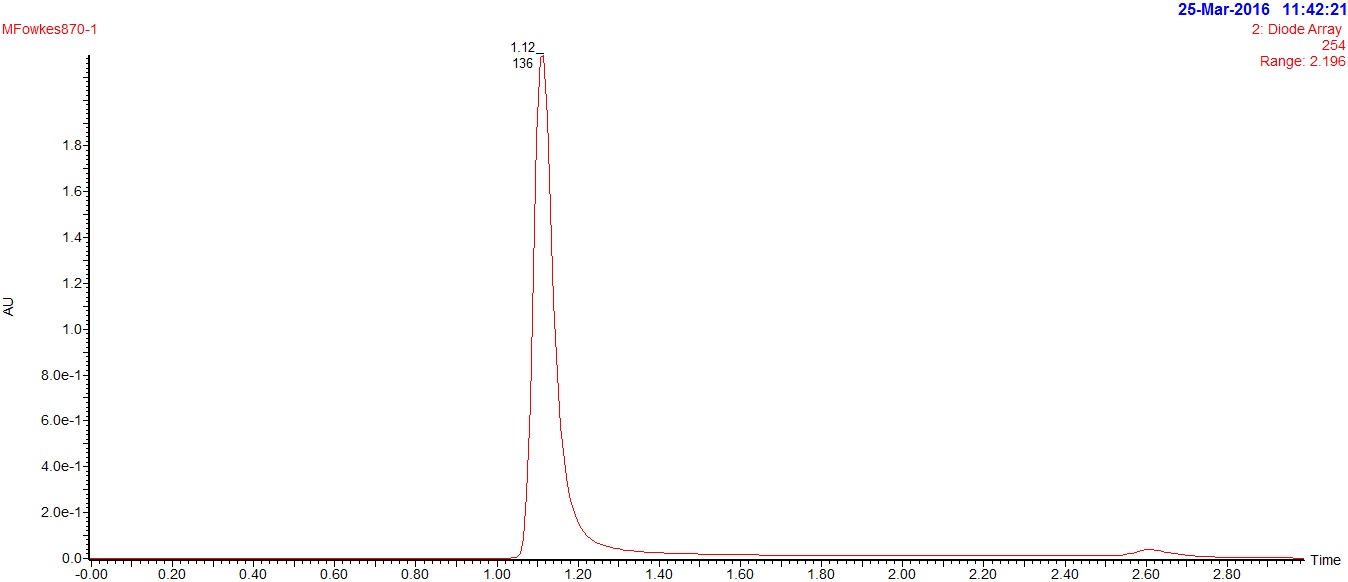
**

### Fig. S2. DIT peptide ^1^H-NMR Spectra

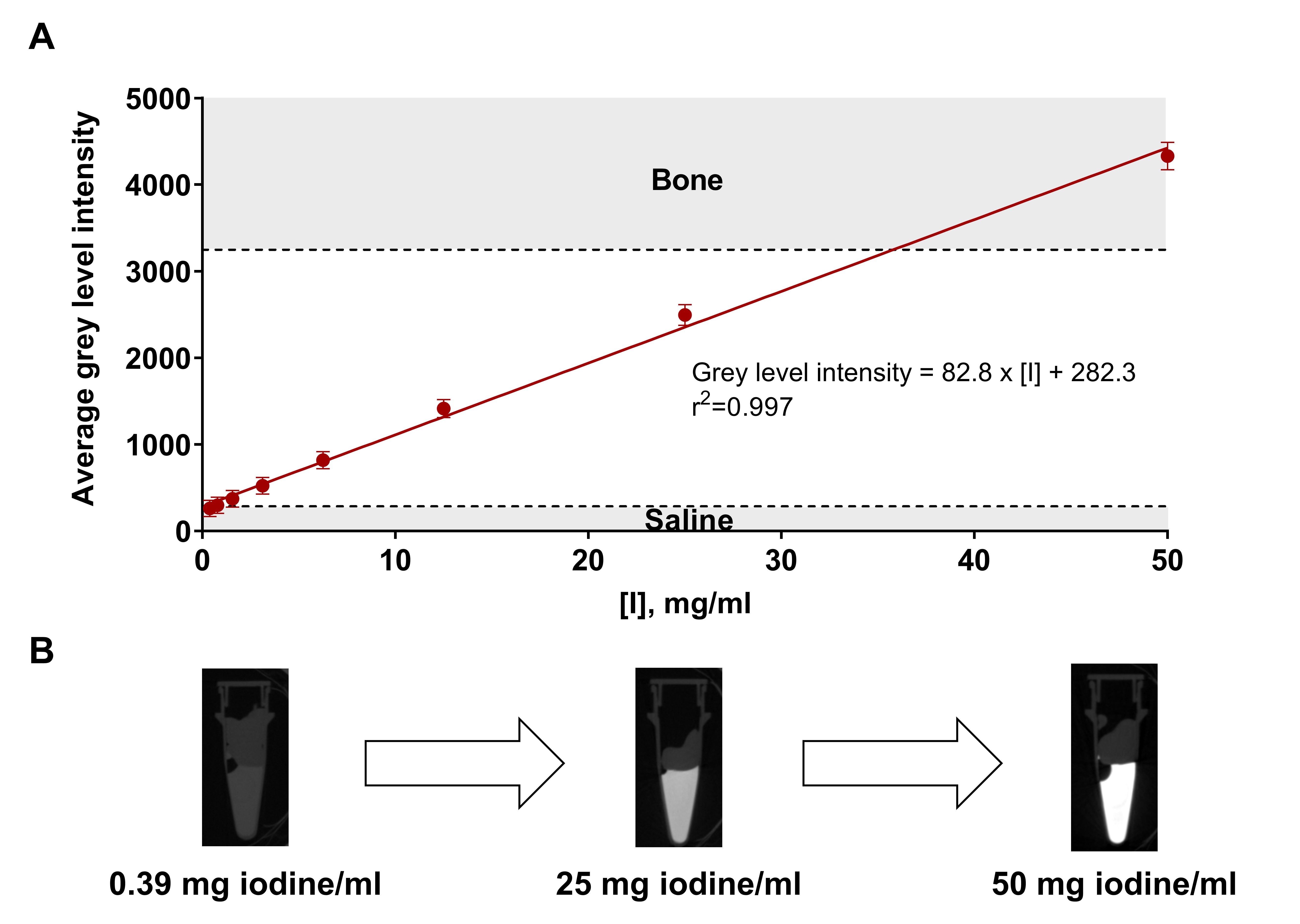


Fig. S3. Concentration of iodine required for effective contrast enhancement of cartilage. (*A*) Iodine calibration curve, showing the linear increase in average grey level intensity with increasing iodine concentration (mean ± SD, *n* = 2). The upper and lower dotted lines denote regions of grey level intensity corresponding to cortical bone and saline, respectively. (*B*) Micro-CT images of three different concentrations of diatrizoic acid, illustrating the linear increase in contrast with increasing iodine concentration.


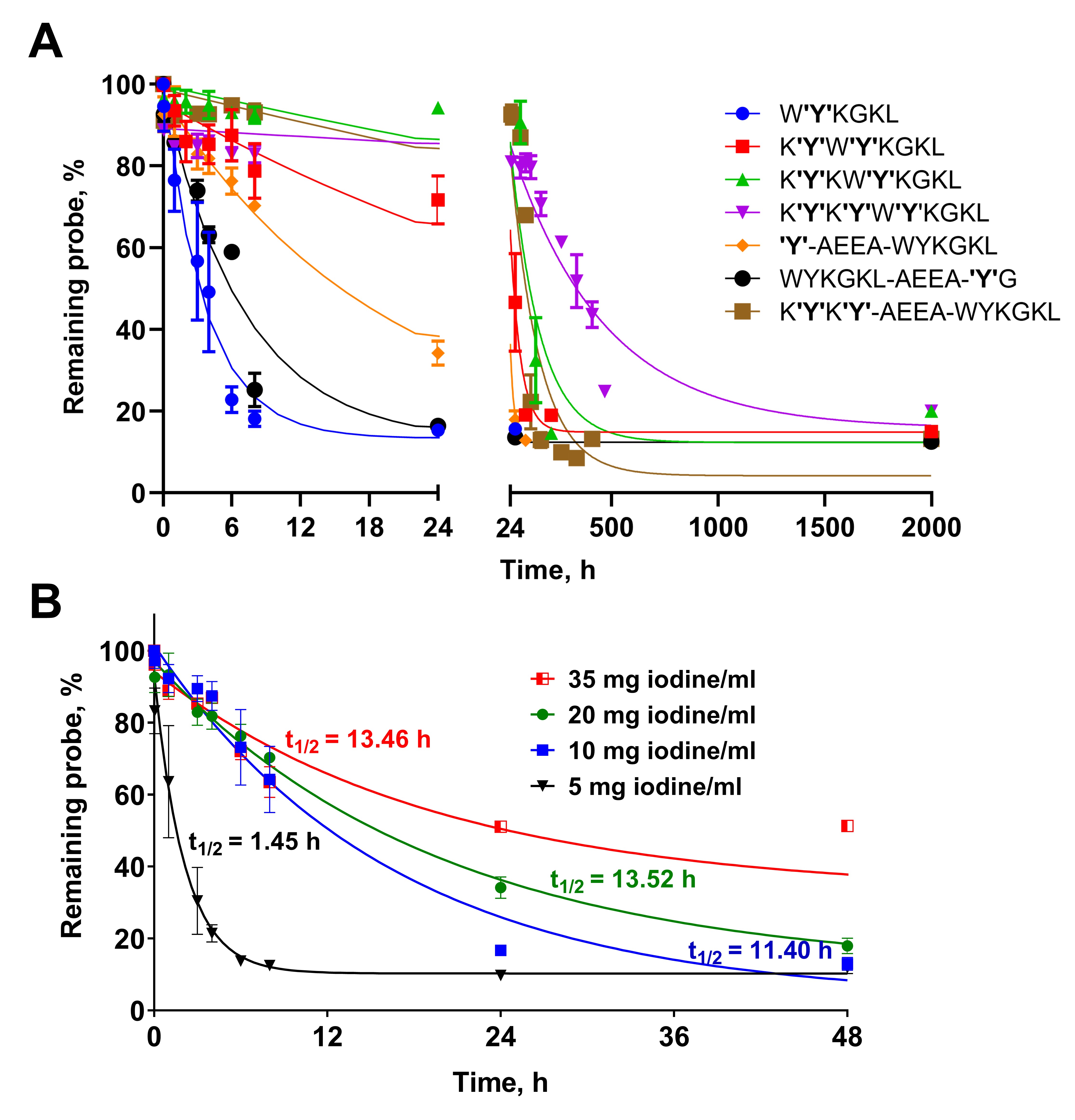


Fig. S4. Curves for DIT peptide radiocontrast agents. (A) Decay curves for DIT peptide radiocontrast agents. Plot of the percentage decrease in contrast over time (mean ± SEM) for DIT peptides binding to cartilage. The half-lives shown in Table 1 were determined through fitting to a model of one-phase exponential decay. (B) Effect of final iodine concentration on the half-life of peptide ˈYˈ-AEEA-WYKGKL (mean ± SEM) after fitting the percentage decrease in contrast over time to a model of one-phase exponential decay.

**
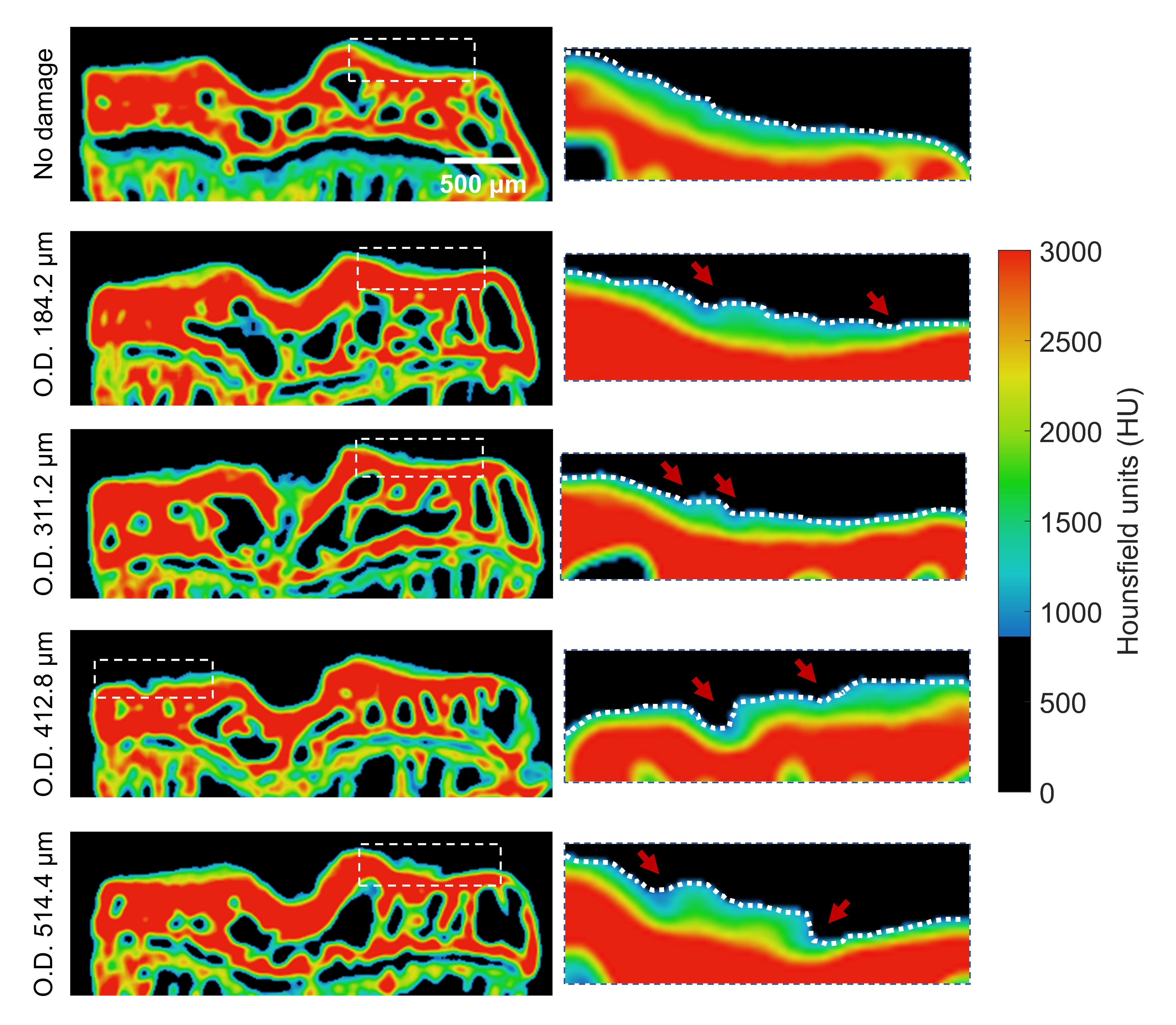
**

Fig. S5*.* *Ex* vivo study of the minimum damage to murine cartilage that DIPIC can detect. Representative colour-coded maps of absorption of tibial coronal views upon incubation to equilibrium with the peptide sequence **ˈYˈ**-*AEEA*-WYKGKL and washing in saline for 1 h. Needles with different outer diameters (O.D.) were used to puncture the surface (red arrows in the dotted boxes) and cartilage subsequently imaged by DIPIC. The dotted white lines delineate the cartilage surface and emphasise the presence of increasingly larger marks in the surface, as opposed to an intact cartilage surface seen in the first row of images.


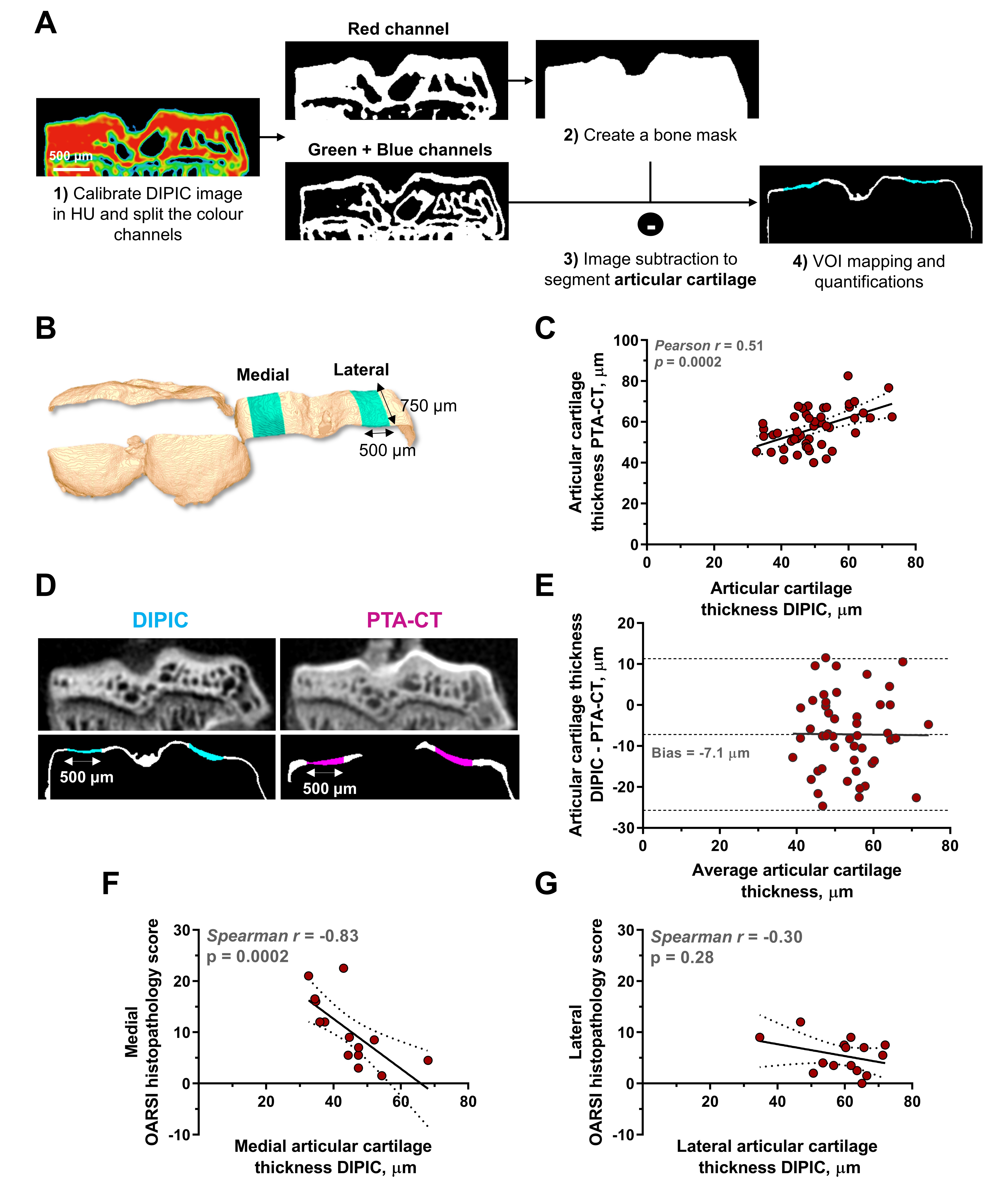


Fig. S6. Image analysis methodology to quantify murine articular cartilage morphology using DIPIC. (*A*) A method based on tissue X-ray absorption was applied to segment articular cartilage and quantify volumes-of-interest (VOIs) in the weight-bearing areas. (*B*) 3D reconstruction of segmented cartilage and corresponding VOIs (light blue) in the medial and lateral aspects of the tibial plateau extending 500 µm in width along 750 µm in the anterior-posterior axis. (*C*) Correlation between cartilage thickness measurements obtained from automated VOIs of DIPIC and PTA-CT; the Pearson correlation coefficient (*r*) and *p*-value are indicated in the graph (*n* = 48). (*D*) Representative and processed micro-CT coronal views of tibiae comparing the automated segmentation and mapping of cartilage between DIPIC and PTA-CT. (*E*) Bland-Altman plot to determine the agreement between DIPIC and PTA-CT measurements of cartilage thickness. The difference between measurements using both methods was plotted against the average (*n* = 48); the bias between methodologies (-7.1 µm) and 95% limits of agreement (-25.6 µm; 11.4 µm) are indicated by dotted lines. Non-parametric correlation between OARSI histopathology scores in the medial (*F*) and lateral (*G*) articular cartilage and medial and lateral articular cartilage thickness measurements obtained by DIPIC (*n* = 15); Spearman correlation coefficients (*r*) and *p*-values are indicated in the graph.

# References

1. Hu HY, Lim NH, Ding-Pfennigdorff D, Saas J, Wendt KU, Ritzeler O, et al. DOTAM derivatives as active cartilage-targeting drug carriers for the treatment of osteoarthritis. Bioconjugate Chemistry. 2015;26(3):383-8. Epub 2015/01/30. doi: 10.1021/bc500557s. PubMed PMID: 25629889.

2. Sole VA, Papillon E, Cotte M, Walter P, Susini J. A multiplatform code for the analysis of energy-dispersive X-ray fluorescence spectra. Spectrochim Acta B. 2007;62(1):63-8. doi: 10.1016/j.sab.2006.12.002. PubMed PMID: WOS:000245367100010.

3. Das Neves Borges P, Forte AE, Vincent TL, Dini D, Marenzana M. Rapid, automated imaging of mouse articular cartilage by microCT for early detection of osteoarthritis and finite element modelling of joint mechanics. Osteoarthritis and Cartilage. 2014;22(10):1419-28. Epub 2014/10/04. doi: 10.1016/j.joca.2014.07.014. PubMed PMID: 25278053; PubMed Central PMCID: PMCPMC4192140.

4. Glasson SS, Blanchet TJ, Morris EA. The surgical destabilization of the medial meniscus (DMM) model of osteoarthritis in the 129/SvEv mouse. Osteoarthritis and Cartilage. 2007;15(9):1061-9. Epub 2007/05/02. doi: 10.1016/j.joca.2007.03.006. PubMed PMID: 17470400.

5. Glasson SS, Chambers MG, Van Den Berg WB, Little CB. The OARSI histopathology initiative - recommendations for histological assessments of osteoarthritis in the mouse. Osteoarthritis and Cartilage. 2010;18:S17-23. Epub 2010/10/01. doi: 10.1016/j.joca.2010.05.025. PubMed PMID: 20864019.
